# Effects of Oligomeric Ultrafine-Nano Hydrogen Water on Laying Performance, Egg Quality, Nutrient Composition, and Intestinal and Reproductive Responses in Late-Laying Hens

**DOI:** 10.3390/ani16172658

**Published:** 2026-08-24

**Authors:** Baowei Wang, Guangpeng Chu, Mengxiao Yang, Zhigang Fan, Yuanzhao Wu, Binghan Wang, Wei Lu, Shijie Fan, Ruilei Liu, Guiqin Wu, Mingai Zhang, Wenlei Fan, Tiejun Chen, Jing Wang

**Affiliations:** 1 College of Food Science and Engineering, Qingdao Agricultural University, Qingdao 266109, China; wangbw@qau.edu.cn (B.W.);; 2 College of Animal Science and Technology, Qingdao Agricultural University, Qingdao 266109, China; 3 Qingdao Londs Technology Equipment Co., Ltd., Qingdao 266109, China; 4 Qingdao Huihe Biotechnology Co., Ltd., Qingdao 266109, China; 5 Beijing Huadu Yukou Poultry Industry Co., Ltd., Beijing 101206, China; 6 Feed Research Institute of Chinese Academy of Agricultural Sciences, Beijing 132011, China

**Keywords:** oligomeric ultrafine-nano hydrogen water, late-laying hens, egg quality, metabolomics, oviduct inflammation, intestinal barrier function

## Abstract

This study evaluated the effects of oligomeric ultrafine-nano hydrogen water (OUHW) on production performance, egg quality, egg nutrient deposition, systemic metabolism, intestinal barrier function, and reproductive status in late-laying hens. Unlike conventional hydrogen-rich water, which primarily delivers molecular hydrogen dissolved in water, OUHW integrates oligomeric water with ultrafine-nano-sized hydrogen bubbles, providing a distinct physicochemical form of hydrogen-water intervention. A total of 288 healthy 67-week-old Jingfen No. 8 laying hens were assigned to 2 treatments with 6 independent replicates per treatment (24 hens per replicate) and provided with either tap water or OUHW for 11 weeks. Compared with the control, OUHW reduced the feed conversion ratio and the rates of manure-spotting while increasing the laying rate, qualified egg rate, and total egg number during weeks 5–8 (*p* < 0.05). These productive and egg quality improvements were phase-specific effects observed in the late laying stage during the 11-week trial. At week 4, OUHW significantly increased egg weight (*p* < 0.05). At week 8, OUHW also improved eggshell compressive elastic deformation, eggshell thickness, albumen height, and Haugh unit (*p* < 0.05). At week 6, eggs from OUHW-treated hens had higher concentrations of glutamate, glycine, alanine, C18:0, C18:1n9c, C20:4n6, and C22:6n3 (*p* < 0.05). Serum metabolomics showed that 25 differential metabolites from the OUHW treatment group were predominantly enriched in glycerophospholipid, sphingolipid, branched-chain amino acid, histidine, and tryptophan metabolism. In the cecum, OUHW decreased p-cresol and increased isovaleric acid and acetic acid (*p* < 0.05). Notably, the overall community structures of the cecal and oviductal microbiota exhibited no significant treatment-related alterations, indicating limited structural changes in the microbial communities in response to the OUHW intervention. OUHW also reduced interleukin-2 and tumor necrosis factor-α, increased interleukin-10 in the jejunum and oviductal isthmus (*p* < 0.05), and upregulated the mRNA expression of ovarian steroidogenic acute regulatory protein and intestinal Occludin, Claudin-1, and Mucin-2 (*p* < 0.05). These findings indicate that OUHW improved laying performance, egg quality, and egg nutrient deposition, possibly through modulation of the metabolism, intestinal barrier function, ovarian function, and oviductal inflammation.

## Simple Summary

Older laying hens often show declining egg production and egg quality, creating challenges for extending the productive lifespan. This study explored whether oligomeric ultrafine-nano hydrogen water could serve as a practical drinking water intervention for late-laying hens. Rather than simply supplying dissolved hydrogen, this water combines small water clusters with ultrafine hydrogen bubbles. Hens receiving this water showed better production efficiency and fewer defective eggs during several periods in the trial, together with improvements in shell and internal egg quality. The treatment also altered the nutritional composition of the eggs and was accompanied by favorable changes in circulating metabolites, intestinal-barrier-related features, cecal fermentation products, and reproductive tract status. These findings indicate that oligomeric ultrafine-nano hydrogen water may support both productivity and physiological resilience in older laying hens. Its use could therefore provide a non-feed-based management approach for maintaining egg production and quality during extended laying cycles, with potential benefits for flock longevity, resource use, and the sustainability of commercial egg production.

## 1. Introduction

Continuous progress in genetic selection, nutrition, and feeding management has substantially extended the productive lifespan of laying hens, with commercial laying cycles now often prolonged to 90 weeks of age or beyond [1]. Extending the laying cycle is economically attractive because it reduces replacement costs, improves resource efficiency, and supports more sustainable egg production [2]. However, maintaining stable laying performance and egg quality during the late laying period remains a major challenge for the poultry industry. After approximately 60 weeks of age, laying hens commonly exhibit a progressive decline in laying rate and egg quality [1,3], which reduces economic returns and limits the practical application of extended-cycle egg production. The decline in egg quality during the late laying period is particularly evident in eggshell and albumen traits [4,5]. The incidence of defective eggs, including sand-spotted, mottled, thin-shelled, cracked, and manure-contaminated eggs, increases with hen age, whereas the albumen height and Haugh unit gradually decrease [1]. These changes impair the commercial value, storage stability, and processing characteristics of eggs. The mechanisms underlying age-related deterioration in egg quality are multifactorial and involve the progressive impairment of reproductive and intestinal functions [6,7]. In the reproductive tract, functional degeneration of the oviduct, especially chronic inflammation and structural impairment of the shell gland or uterus, may compromise ion transport, matrix secretion, and mineral deposition, thereby impairing eggshell formation and calcification [8]. Intestinal barrier dysfunction, villus atrophy, and impaired tight junction integrity may compromise nutrient digestion and absorption, thereby limiting the availability of amino acids, calcium, phosphorus, and other nutrients essential for egg formation [6,9]. Moreover, increased oxidative stress and impaired antioxidant defenses in aged laying hens may exacerbate intestinal and reproductive tissue damage, thereby contributing to age-related functional decline [10,11]. Therefore, nutritional strategies that can alleviate oxidative stress and inflammation, improve intestinal barrier function, and support oviductal and ovarian function are of practical importance for late-laying hens.

Hydrogen-rich water (HRW) has attracted increasing attention as a functional drinking water because of its potential antioxidant, anti-inflammatory, and metabolic regulatory properties, which have mainly been reported in humans and mice [12,13]. Molecular hydrogen has been reported to selectively reduce excessive reactive oxygen species and regulate redox-sensitive signaling pathways. Previous studies have suggested the antioxidant effects of electrolyzed HRW with reduced oxidative stress demonstrated in stressed rats [14]; more context-dependent antioxidant responses were observed in patients with type 2 diabetes mellitus [15]. In poultry, HRW at 1200 ppb enhanced antioxidant status in broilers [16]. These findings suggest that hydrogen-based drinking water may be a promising approach for improving physiological resilience and production performance in poultry. However, conventional HRW is limited by rapid hydrogen dissipation, low hydrogen retention, and poor storage stability [17], which may restrict its biological efficacy under practical production conditions.

Oligomeric ultrafine-nano hydrogen water (OUHW) is prepared using electrolysis-driven hydrogen production followed by an ultrafine-nano bubble generation process, which facilitates the dispersion and retention of hydrogen in water [18]. Ultrafine hydrogen bubbles exhibit prolonged persistence in aqueous systems, and ultrafine-bubble hydrogen water has been reported to show improved storage stability compared with conventionally dissolved hydrogen water [17,19]. These physicochemical characteristics may allow more sustained hydrogen availability. Previous studies have shown that hydrogen nanobubble water or hydrogen-rich water can attenuate oxidative stress and inflammatory responses and influence metabolic processes in experimental models [18,20]. These properties make OUHW a potential intervention for mitigating age-related intestinal barrier impairment, oviductal inflammation, and reproductive decline in late-laying hens. Nevertheless, to our knowledge, no study has evaluated the effects of OUHW in late-laying hens, and its potential roles in regulating egg production, egg quality, intestinal and oviductal health, and systemic metabolism remain unclear.

Previous work showed that oligomeric ultrafine-nano hydrogen water (OUHW) improved flock uniformity, antioxidant capacity, intestinal development, and cecal microbial and metabolic profiles in growing layer-type chickens. However, its effects on reproductively active, aging laying hens remain unclear. Therefore, this study evaluated the effects of OUHW on laying performance, egg quality, egg nutrient composition, systemic and intestinal metabolism, intestinal barrier function, and reproductive status in late-laying hens. We hypothesized that improvements in laying performance and egg quality would be associated with favorable metabolic, intestinal, and reproductive responses. This study may provide further evidence for the potential use of OUHW as a functional drinking water strategy in extended-cycle laying hen production.

## 2. Materials and Methods

### 2.1. Animal Ethics Statement

All experimental procedures involving animals were conducted in accordance with the guidelines for the care and use of agricultural animals in research and were approved by the Institutional Animal Care and Use Committee of Qingdao Agricultural University on 28 April 2024 (Approval No. DKY20240428). Humane treatment was provided, pain reduced, and the 3R principles followed, ensuring scientific validity and rational animal use.

### 2.2. Experimental Design and Bird Management

The feeding trial was conducted at Shandong Yukou Poultry Co., Ltd. (Jining, China). A total of 288 healthy 67-week-old Jingfen No. 8 laying hens with similar body weight and laying performance were used in this study. Hens were randomly allotted to 2 treatments with 6 replicates per treatment and 24 hens per replicate. Each replicate contained six cages under a four-tier full-step cage-housing system, with four hens housed per cage. Both groups shared identical-source tap water; the control group received untreated tap water, whereas the OUHW group received oligomeric ultrafine-nano hydrogen water processed from the same source of tap water. The experiment lasted 11 weeks, from 67 to 77 weeks of age.

The trial was conducted at Shandong Yukou Poultry Industry Co., Ltd. (Jining, China). Birds were maintained under standard commercial management conditions in a four-layer full-step cage housing system, with four hens raised per cage. The indoor temperature of the henhouse was maintained at 26 ± 2 °C, and the relative humidity was kept at 55–65% throughout the trial period. The lighting program was set as a 16 h light: 8 h dark cycle during the entire experimental period. Feed was supplied manually, and feed was available ad libitum for all birds. All experimental hens were reared under standard management conditions and received routine vaccinations according to the recommended vaccination schedule for laying hens. The experimental period lasted 11 weeks, after which the birds were humanely euthanized for sample collection.

### 2.3. Preparation and Characterization of OUHW

OUHW was prepared using an ultrafine-nano hydrogen water generation system (model LZ-2000; Qingdao Langzi Technology Equipment Co., Ltd., Qingdao, China). Fresh OUHW was supplied to hens at regular intervals each day to maintain hydrogen availability during the experimental period. During the trial, both the control and treatment groups had ad libitum access to water via nipple drinkers throughout the day and night, and the water lines were automatically flushed once per week.

The physicochemical properties of OUHW were determined before use. The drinking water quality in both the control and treatment groups met the requirements of the relevant national standards. The main physicochemical and microbiological parameters for both water sources were determined as follows: total dissolved solids (TDS) at 1850 mg/L, pH at 7.2, oxidation-reduction potential (ORP) at +210 mV, total coliforms not detected, and no residual disinfectant was detected in either group. The half-width value measured via nuclear magnetic resonance (AVANCE 500, Bruker, Berlin, Germany) was 52.08 Hz. The NMR half-width reflects the size of water molecular clusters; smaller values facilitate passage through cellular aquaporins (AQP), thereby enhancing bioavailability. For the control tap water, the ^17^O NMR half-width was approximately 100–120 Hz. Surface tension decreased from 70.94 to 61.06 mN/m after treatment. The particle size of the micro- and nanobubbles ranged from 30 to 60 nm. Hydrogen purity was 99.53%, and the initial dissolved hydrogen concentration was 1384 ppb, which remained at approximately 1130 ppb after 8 h. Dissolved hydrogen concentrations were further measured at the drinker outlet (point of consumption) before the trial and at the mid-point of the trial. The average value was 900 ppb, which, although slightly lower than the initial value (>1000 ppb), remained at a relatively high level, ensuring the effective intake of active hydrogen by the laying hens. Major physicochemical parameters, including hydrogen concentration, bubble size distribution, hydrogen retention time, half-width, and surface tension, met the technical requirements of this standard. The adopted group standard is T/QDIFST 007-2025 Oligomeric Ultra-nano Hydrogen Water. Its key acceptance limits for core functional-water parameters are as follows: ultra-nano bubble diameter in the range of 30–60 nm, accounting for ≥85% of total bubbles, hydrogen concentration ≥ 1000 ppb maintained for over 48 h, ^17^O-NMR half-width ≤ 85 Hz (for oligomeric water ≤ 60 Hz), and the basic quality of the source water must comply with GB 5749 Standards for Drinking Water Quality. The physicochemical properties of OUHW were measured before the trial and at the mid-point of the trial to ensure that the water used in the experiment met the required standards.

### 2.4. Diet

All hens were fed the same basal diet throughout the experiment. The basal diet was formulated to satisfy nutrient requirements specified in the commercial feeding manual for Jingfen No. 8 laying hens. The ingredient composition and nutrient levels of the basal diet are shown in Table 1. Metabolizable energy was calculated from feed composition, whereas crude protein, calcium, phosphorus, and amino acids were analyzed chemically.

### 2.5. Production Performance

Egg production, egg weight, and the numbers of breaking, manure-stained, and pimpled eggs were recorded daily for each replicate. Production parameters were calculated on a replicate basis according to commonly used procedures for laying hens [21,22,23]. Total egg number was calculated as the cumulative number of eggs produced by all hens within each replicate during the corresponding experimental period. Laying rate (%) was calculated as the total number of eggs produced divided by the total number of hen-days and multiplied by 100. The egg-breaking rate (%) was calculated as the number of broken eggs divided by the total number of collected eggs within the observation period, multiplied by 100. The average egg weight (g/egg) was calculated by dividing the total weight of eggs collected by the corresponding number of eggs. The feed conversion ratio (FCR; g feed/g egg) was calculated as total feed intake divided by total egg mass [23].

Egg defects were evaluated via visual inspection following previously reported classifications of defective eggs [21,22]. Eggs with visible shell fractures were classified as cracked eggs, whereas eggs with visible manure contamination on the shell surface were classified as manure-stained eggs. Pimpled eggs were identified by the presence of abnormal, raised, extra-calcified granules or pimple-like deposits on the eggshell surface, as described previously [24]. The cracked egg rate, manure-stained egg rate, and pimpled egg rate were calculated as the number of eggs in each respective defect category divided by the total number of eggs produced and multiplied by 100. The egg-weight-graded proportions (45–52 g rate, 52–60 g rate, and 60–65 g rate, %) were calculated as the number of eggs within each respective weight range divided by the total number of collected eggs, multiplied by 100. The qualified egg rate (%) was calculated as the number of normal qualified eggs (excluding manure-spotted eggs, pimpled eggs, and broken eggs) divided by the total number of collected eggs, multiplied by 100.

Daily observations were summarized weekly for each replicate. For statistical analysis, the 11-week experimental period was further divided into three phases: weeks 1–4, weeks 5–8, and weeks 9–11, and cumulative values were also calculated for the entire experimental period where applicable.

### 2.6. Egg Quality Measurements

Egg quality was evaluated at weeks 4, 8, and 11. At each sampling time point, 5 externally normal eggs with intact shells were randomly selected from each replicate (30 eggs per treatment) for the standardized measurement of conventional egg quality traits. Importantly, visible egg abnormalities were not omitted from the overall evaluation, as the broken egg rate, manure-spotted egg rate, and qualified egg rate were recorded separately as production performance indicators, together with the laying rate. Eggs accidentally damaged during sampling or handling were excluded from the egg quality measurements because such damage was unrelated to the experimental treatment.

Egg quality traits included egg weight, eggshell color, eggshell gloss, eggshell compressive elastic deformation, eggshell strength, eggshell brittle deformation, albumen height, yolk color, Haugh unit, eggshell weight, yolk weight, eggshell percentage, yolk percentage, eggshell thickness, and egg shape index.

Eggshell compressive elastic deformation, eggshell strength, and eggshell brittle deformation were measured using a Type-II eggshell strength tester (force-sensor range: 5 kg, test speed: 2 mm/s; Robotmation, Tokyo, Japan). In the present study, the term “eggshell mechanical deformation indices” collectively includes eggshell compressive elastic deformation, deformation under 9.8 N, eggshell brittle-fracture deformation, and eggshell strength. Eggshell thickness was measured at the blunt end, equator, and sharp end using a wall-tube micrometer (Ozaki Seisakusho, Tokyo, Japan), and the average value was calculated as overall eggshell thickness. Eggshell percentage and yolk percentage were calculated according to the ratios of eggshell weight and yolk weight to total egg weight using an analytical balance (BSA224S-CW, Sartorius, Göttingen, Germany). Eggshell color parameters (L*, a*, b*) were determined using a color difference meter (CR-400, Konica Minolta, Tokyo, Japan), and eggshell gloss was determined using a glossmeter (HG268, 3nh, Shenzhen, China). Albumen height, yolk color, and calculated Haugh unit were obtained using a multifunctional egg quality analyzer (ETU-01, ORKA Food Technology Ltd., Ramat Hasharon, Israel) according to the manufacturer’s instructions. Egg shape index and egg weight were also assessed using this multifunctional egg quality analyzer.

### 2.7. Determination of Egg Amino Acids and Fatty Acids

At week 6 (mid-trial) and week 11 (end-of-trial), 2 eggs were randomly collected from each replicate for amino acid and fatty acid analyses. These two time points were chosen to evaluate the cumulative effects of long-term OUHW supplementation on egg nutrient deposition. The contents of the two eggs from the same replicate were pooled and thoroughly homogenized to prepare one composite sample per replicate at each sampling time. The homogenate was then divided into separate aliquots for amino acid and fatty acid analyses.

Amino acid composition was determined using an automatic amino acid analyzer (LA8080 AminoSAAYA, Hitachi High-Tech Corporation, Tokyo, Japan). For amino acids other than sulfur-containing amino acids, samples were hydrolyzed with 6 M HCl at 110 °C for 24 h, followed by ion-exchange chromatography and post-column ninhydrin derivatization. For methionine and cysteine determination, a separate aliquot was pre-oxidized with performic acid at 0 °C for 16 h to convert methionine and cysteine to methionine sulfone and cysteic acid, respectively. Excess performic acid was decomposed using sodium metabisulfite, followed by acid hydrolysis as described above. Methionine was quantified as methionine sulfone, whereas cysteine was quantified as cysteic acid and converted to cysteine equivalents for reporting [25]. Flavor amino acids were defined as the sum of umami amino acids (aspartic acid and glutamic acid) and sweet amino acids (serine, glycine, alanine, and proline) [26]. Total flavor amino acid content was expressed as the sum of these 6 amino acids (g/100 g).

Fatty acid composition was determined via gas chromatography (GC-2010, Shimadzu Corporation, Kyoto, Japan) using an HP-88 capillary column after lipid extraction and fatty acid methyl ester preparation. The oven temperature was maintained at 100 °C for 15 min, increased to 190 °C at 20 °C/min and held for 6 min, and then increased to 220 °C at 1 °C/min and held for 7 min. The injector and flame-ionization detector temperatures were 260 and 250 °C, respectively. Amino acid and fatty acid contents were expressed on a sample-weight basis.

### 2.8. Sample Collection

At the end of the 11-week trial, one hen from each replicate was selected using simple random sampling, with each hen within the replicate having an equal probability of being selected (*n* = 6 per treatment). Hen selection was not based on baseline laying performance because laying performance was recorded and evaluated at the replicate level rather than being used as an individual-level selection criterion. To minimize variation in circulating reproductive hormone concentrations associated with the ovulatory cycle, blood samples were collected at a standardized time relative to oviposition. Blood samples were collected from the wing vein, allowed to clot at room temperature, and centrifuged to obtain serum, which was stored at −80 °C for metabolomics analysis.

After blood collection, hens were humanely euthanized via manual cervical dislocation performed by trained personnel in accordance with the AVMA Guidelines for the Euthanasia of Animals [27], and death was confirmed by the absence of rhythmic breathing and reflexes.

After euthanasia, approximately 2 cm segments of the duodenum, jejunum, and ileum were collected, gently flushed with sterile saline, and fixed in 4% paraformaldehyde for histomorphological analysis. In addition, jejunal mucosa, cecal contents, ovarian tissue, and oviductal tissues from the ampulla, isthmus, and uterus were collected. The jejunal mucosa was gently scraped with sterile instruments after rinsing the jejunal segment with sterile saline. Cecal contents were aseptically collected from both ceca, and oviductal mucosal secretions were collected under sterile conditions. Samples for molecular analysis, metabolite determination, and microbiota analysis were immediately frozen in liquid nitrogen and stored at −80 °C until analysis.

### 2.9. Histomorphological Evaluation

Tissue segments fixed in 4% paraformaldehyde were maintained at room temperature for 72 h before being processed for paraffin embedding, sectioning at 5 μm thickness (RM2235 microtome; Leica Biosystems, Wetzlar, Germany), and staining with hematoxylin–eosin (HE) by Qingdao Standard Testing Co., Ltd. (Qingdao, China). Intestinal villus height (VH) and crypt depth (CD) were measured using a digital image analysis system (BX53 microscope with DP74 camera; Olympus Corp., Tokyo, Japan), and the VH/CD ratio was calculated. For each bird, one representative tissue section from each intestinal segment was evaluated. Multiple non-overlapping microscopic fields across the section were examined to identify suitable structures; the number of fields was not fixed because fields were screened until 10 intact and well-oriented villi and their corresponding 10 crypts had been identified. Thus, a total of 10 villi and 10 corresponding crypts were measured per intestinal segment per bird. The mean value of these 10 measurements was used as the experimental value for statistical analysis. Histomorphometric measurements were performed by an observer blinded to the treatment allocation. The heights of the primary and secondary mucosal folds in the magnum, the mucosal fold height in the isthmus, and the mucosal fold spacing in the uterus were measured using a digital image analysis system (BX53 microscope with DP74 camera; Olympus Corp., Tokyo, Japan).

### 2.10. Serum Biochemical, Reproductive Hormonal, and Metabolomics Analysis

Thawed serum samples were used to analyze a comprehensive suite of physiological markers. Antioxidant indices (MDA, SOD, T-AOC, GSH-Px, CAT, and GSH) and immune-inflammatory factors (IgG, IgA, IgM, IL-6, TNF-α, IL-1β, IL-10) were measured using ELISA commercial kits (Shanghai Tongwei Biological Technology Co., Ltd., Shanghai, China). To characterize the systemic reproductive status, serum levels of estradiol (E_2_), follicle-stimulating hormone (FSH), luteinizing hormone (LH), and progesterone (PROG) were also determined via ELISA commercial kits (Shanghai Tongwei Biological Technology Co., Ltd., China). All commercial ELISA kits used in this study have been specifically validated and confirmed to be applicable for avian samples. Detailed technical parameters for each kit, including catalogue number, detection range, sensitivity, intra-assay coefficient of variation (CV) and inter-assay CV, are listed in Table S1 for reference.

Serum samples preserved at −80 °C were thawed at room temperature. An aliquot of 100 μ was transferred into a 1.5 mL centrifuge tube, mixed with 20 μL of the internal standard L-2-chlorophenylalanine (0.06 mg/mL methanol), and vortexed for 10 s. Subsequently, 300 μL of pre-cooled methanol/acetonitrile extractant (2:1, *v*/*v*) was added, and the mixture was vortexed for 1 min. The samples were sonicated in an ice-water bath for 10 min, incubated at −20 °C for 30 min, and centrifuged at 13,000× *g* for 10 min at 4 °C. A 200-μL aliquot of the resulting supernatant was collected and evaporated to dryness. The dried extract was reconstituted in 300 μL of methanol/water (1:4, *v*/*v*), vortexed for 30 s, sonicated in an ice-water bath for 3 min, and incubated at −20 °C for 2 h. After a second centrifugation at 13,000× *g* for 10 min at 4 °C, 150 μL of the supernatant was collected, filtered through a 0.22 μm membrane filter, and stored at −80 °C until LC–MS analysis.

Pooled quality control (QC) samples were prepared by mixing all sample aliquots. Metabolomic profiling was conducted using an ACQUITY UPLC I-Class plus system coupled with a Q-Exactive mass spectrometer (Thermo Fisher Scientific, Waltham, MA, USA) under dual ESI positive and negative modes, with an ACQUITY UPLC HSS T3 column (1.8 μm, 2.1 mm × 100 mm; Waters Corporation, Milford, MA, USA). Mobile phase A was 0.1% formic acid aqueous solution (*v*/*v*), and mobile phase B was 0.1% formic acid acetonitrile solution (*v*/*v*); the flow rate was set to 0.35 mL/min and the column temperature maintained at 45 °C. The gradient elution program was as follows: 0.01 min, 5% B; 2 min, 5% B; 4 min, 30% B; 8 min, 50% B; 10 min, 80% B; 14 min, 100% B; 15 min, 100% B; 15.1–16 min, 5% B. The autosampler temperature was kept at 10 °C throughout detection. Full MS scans covered *m*/*z* 100–1000 at a resolution of 70,000, and data-dependent HCD MS/MS spectra were acquired at a 17,500 resolution with normalized collision energies of 10, 20, and 40 eV. Ion source parameters were configured as: spray voltage, 3800 V (ESI+)/3200 V (ESI−); sheath gas flow, 35 arbitrary units; auxiliary gas flow, 8 arbitrary units; capillary temperature, 320 °C; auxiliary gas heater temperature, 350 °C; S-lens RF level, 50.

Raw LC-MS data were processed using Progenesis QI V2.3 software (Nonlinear Dynamics, Newcastle, UK). Metabolite identification criteria were predefined: precursor mass error tolerance of 5 ppm, fragment ion mass error tolerance of 10 ppm, and retention-time tolerance of ±0.3 min for database matching. Compound matching scores ranged from 0 to 60, and only metabolites with an identification score ≥ 36 were retained as reliable annotations. All metabolites were annotated against HMDB, Lipidmaps (V2.3), Metlin, and an in-house standard library, before being further categorized into four confidence levels (Level 1–Level 4) according to MSI-based metabolite identification standards.

After data cleaning, integrated ion matrices were analyzed in R via PCA, PLS-DA and OPLS-DA models. To evaluate the potential over-fitting of the supervised OPLS-DA model (*n* = 6 per group), 7-fold cross-validation and 200-response permutation testing were performed. The cumulative model parameters were R^2^Y = 0.994 and Q^2^ = 0.352. The permutation test showed the intercept of the permuted −Q^2^ regression line was above zero, implying a risk of model overfitting. Therefore, differential metabolites were screened using a strict dual threshold (VIP > 1.0 combined with *p* < 0.05), rather than relying solely on the OPLS-DA model output. Subsequent KEGG pathway enrichment analysis was performed for differential metabolites.

### 2.11. Determination of Cecal Metabolites

Cecal contents were analyzed for short-chain fatty acids (SCFAs), ammonia nitrogen, and potentially harmful aromatic microbial metabolites. SCFAs, including acetic acid, propionic acid, n-butyric acid, isobutyric acid, n-valeric acid, isovaleric acid, and hexanoic acid were determined via gas chromatography with flame ionization detection (GC-FID) as previously described [28], with minor modifications. Briefly, frozen cecal samples were thawed on ice, homogenized with ultrapure water, acidified with 25% metaphosphoric acid containing crotonic acid as an internal standard, and centrifuged at 12,000× *g* for 15 min at 4 °C. The supernatants were filtered through a 0.22 μm membrane and analyzed using a GC-2014 gas chromatograph (Shimadzu Corporation, Kyoto, Japan) equipped with a DB-FFAP capillary column (30 m × 0.25 mm × 0.25 μm) and a FID. The injector and detector temperatures were 220 and 250 °C, respectively. The oven temperature was initially maintained at 60 °C for 2 min, increased to 120 °C at 10 °C/min and then to 200 °C at 15 °C/min, and held for 3 min. Nitrogen was used as the carrier gas at 1.0 mL/min with a split ratio of 10:1. SCFAs were identified by comparison with authentic standards and quantified using the internal standard method.

Ammonia nitrogen was determined using the salicylate–hypochlorite colorimetric method [28]. Cecal contents were extracted with 5% lithium carbonate solution and centrifuged at 10,000× *g* for 15 min at 4 °C. The supernatants were reacted with salicylate and hypochlorite reagents for 30 min at room temperature, and absorbance was measured at 685 nm.

Phenol, p-cresol, indole, and 3-methylindole were determined by GC-FID following liquid–liquid extraction, as described by Cho et al. [29], with minor modifications. The cecal extract was mixed with an equal volume of chloroform under alkaline conditions, vortexed, and centrifuged. The chloroform phase was filtered through a 0.22-μm organic membrane before GC analysis. Metabolites were identified using authentic standards and quantified using corresponding calibration curves. All metabolite concentrations are expressed as mg/kg of wet cecal contents.

### 2.12. RNA Extraction and Quantitative Real-Time PCR

Total RNA was extracted from the frozen ileal mucosa and ovarian tissues using Trizol reagent (Invitrogen, Carlsbad, CA, USA) according to the manufacturer’s protocol. RNA concentration and purity were assessed using a NanoDrop 2000 spectrophotometer (Thermo Fisher Scientific, Waltham, MA, USA). The relative mRNA expression of key genes was determined via quantitative real-time PCR (qRT-PCR) by Qingdao Standard Testing Co., Ltd. Target genes included intestinal barrier markers (*ZO-1*, *Occludin*, *Claudin-1*, *E-cadherin*, and *Mucin-2*) and ovarian functional markers, specifically those related to apoptosis (*p53* and *CASP3*) and steroidogenesis (*StAR* and *FSHR*). qPCR was performed in a total reaction volume of 20 μL, containing 10 μL of 2× SYBR Green qPCR Master Mix, 0.8 μL each of forward and reverse primers (0.4 μM final concentration for each primer), 2 μL of cDNA template, and nuclease-free water to the final volume. The primer sequences used in the experiments are listed in Table 2. The reaction program was set as follows: initial pre-denaturation at 95 °C for 5 min; followed by 40 amplification cycles consisting of denaturation at 95 °C for 10 s, annealing and extension at 60 °C for 30 s (fluorescence signal was collected at the annealing and extension step). The melting curve analysis was performed by heating from 60 °C to 95 °C at a ramp rate of 0.05 °C/s with continuous fluorescence acquisition. The amplification efficiencies of the target genes and GAPDH were comparable, supporting the use of the 2^−ΔΔCt^ method. Each biological sample was analyzed in [duplicate/triplicate] technical reactions, and the mean Ct value was used for subsequent calculations. A no-template control (NTC), in which cDNA was replaced with nuclease-free water, was included for each primer pair in each qPCR run, and no specific amplification was detected. The expression stability of GAPDH as a single reference gene was validated across all experimental samples in the present study. GAPDH was used as the internal control, and relative expression levels were calculated using the 2^−ΔΔCt^ method.

### 2.13. Determination of Inflammatory Cytokines and Immune-Related Indicators

The oviductal isthmus and jejunal mucosa were used to determine inflammatory cytokines and immune-related indicators. Tissue samples were homogenized in an ice-cold buffer and centrifuged to obtain the supernatant. Protein concentration in the supernatant was determined using the bicinchoninic acid method.

The concentrations of cytokines and immune-related factors were measured using commercial ELISA kits (Shanghai Tongwei Biological Technology Co., Ltd., China) according to the manufacturers’ instructions. In the oviductal isthmus, IL-6, TNF-α, IL-1β, IL-10, IFN-γ, and IL-2 were determined. In the jejunal mucosa, IL-6, TNF-α, IL-1β, IL-10, IFN-γ, IL-2, polymeric immunoglobulin receptor (PIGR), and secretory immunoglobulin A (slgA) were measured using commercial ELISA kits (Shanghai Tongwei Biological Technology Co., Ltd., China). All commercial ELISA kits used in this study have been specifically validated and confirmed to be applicable for avian samples. Detailed technical parameters for each kit, including catalogue number, detection range, sensitivity, intra-assay coefficient of variation (CV) and inter-assay CV, are listed in Table S1 for reference. The results were normalized to the tissue protein concentration where appropriate.

### 2.14. Analysis of Intestinal and Oviduct Microbiota

Microbial DNA was extracted from cecal contents and oviduct mucosal secretions using a commercial microbial DNA extraction kit (M5635-02; Qiagen, Hilden, Germany) according to the manufacturer’s instructions. The concentration and purity of the extracted DNA were assessed using a NanoDrop 2000 spectrophotometer (Thermo Fisher Scientific, Waltham, MA, USA). Specifically, extraction blanks consisting of nuclease-free water without biological material were processed in parallel with the biological samples throughout the entire DNA extraction procedure to monitor potential contamination originating from extraction reagents and laboratory handling. The V3–V4 region of the 16S rRNA gene was amplified using primers 341F (5′-CCTACGGGNGGCWGCAG-3′) and 806R (5′-GGACTACHVGGGTATCTAAT-3′). Amplicons were purified, quantified, pooled in equimolar amounts, and sequenced on an Illumina NovaSeq 6000 platform (Illumina Inc., San Diego, CA, USA). Library preparation and high-throughput sequencing, together with subsequent raw data processing, were completed by OE Biotech Co., Ltd. (Shanghai, China). PCR no-template controls (NTCs), in which template DNA was replaced with nuclease-free water, were included in each amplification batch to monitor contamination introduced during PCR preparation and amplification. In addition, environmental/sampling controls, consisting of sterile swabs exposed to the sampling environment and handled using the same procedures as the oviduct samples, but without contact with biological material, were included to assess potential contamination introduced during sample collection and handling. Raw sequencing outputs were generated in FASTQ format. Cutadapt software (version 2020.11) was used to remove sequencing adapters from paired-end raw reads. Quality trimming, low-quality sequence filtering, denoising, read merging, and chimera removal were performed via the DADA2 plugin under the default parameters of QIIME 2 (version 2020.11), generating representative sequences and an amplicon sequence variant (ASV) abundance table. Taxonomic annotation of all representative ASV sequences was implemented in the QIIME 2 pipeline using the q2-feature-classifier with default settings against the Silva reference database (release 138).

Microbial α-diversity indices including Good’s-coverage, Chao1, and Shannon indices were calculated to evaluate community coverage, richness and diversity. For β-diversity analysis, PERMANOVA tests based on the Bray–Curtis distance and UPGMA hierarchical clustering analysis were performed to assess differences in microbial community structure between groups. Taxonomic composition was analyzed at the phylum and genus levels using QIIME 2 software to identify changes in cecal and oviduct microbial profiles associated with OUHW treatment.

### 2.15. Statistical Analysis

Statistical analyses of conventional phenotypic data were performed using SPSS 26.0 (IBM Corp., Armonk, NY, USA). For production performance, the replicate was considered the experimental unit. For egg quality, egg amino acid and fatty acid composition, cecal metabolites, gene expression, inflammatory and immune indices, the sampled egg, individual bird, or biological sample was considered the experimental unit, as appropriate. Data were assessed for normality using the Shapiro–Wilk test and for homogeneity of variance using Levene’s test. For normally distributed data with homogeneous variances, differences between the control and OUHW groups were evaluated using an independent-samples *t*-test. Differences were considered statistically significant at *p* < 0.05. Except for gene expression data, which are presented as means ± SD, all other conventional data are presented as means and SEM. Differences were considered significant at *p* < 0.05.

For serum metabolomics, normalized metabolite data were subjected to principal component analysis (PCA) to evaluate overall metabolic variation and orthogonal partial least-squares discriminant analysis (OPLS-DA) to assess metabolic differences between treatments. The robustness of the OPLS-DA model was evaluated using permutation testing. Differential metabolites were identified based on a variable importance in projection (VIP) value > 1.0 and *p* < 0.05 from univariate analysis. Differential metabolites were subsequently mapped to the Kyoto Encyclopedia of Genes and Genomes (KEGG) database for metabolic pathway enrichment analysis.

Microbiota data were analyzed separately using QIIME 2. Alpha diversity was assessed using the Chao1 richness and Shannon diversity indices, and differences between treatments were evaluated using the Wilcoxon rank-sum test. Beta diversity was assessed by PERMANOVA based on Bray–Curtis distance and UPGMA hierarchical clustering analysis were performed to assess differences in microbial community structure between groups. Differentially abundant taxa between treatments were identified using linear discriminant analysis effect size (LEfSe), with *p* < 0.05 and an LDA score ≥ 2.0 considered significant.

## 3. Results

### 3.1. Production Performance

The average water intake of laying hens during the late laying period was 300–400 mL per hen per day in both the control and treatment groups, with no significant difference between the two groups, indicating that OUHW treatment did not affect water palatability. The effects of OUHW on production performance are shown in Table 3. During weeks 1 to 4, hens in the OUHW group had a lower FCR and a higher laying rate and proportion of 45–52 g eggs than those in the control group (*p* < 0.05). During weeks 5 to 8, OUHW decreased FCR, the manure-stained egg rate, and the pimpled egg rate (*p* < 0.05), and increased the total egg number, proportion of 45–52 g eggs, qualified egg rate, and laying rate (*p* < 0.01). During weeks 9 to 11, FCR, the manure-stained egg rate, and the cracked egg rate were lower in the OUHW group than in the control group (*p* < 0.05), whereas the total egg number, proportion of 52–60 g eggs, and laying rate were higher (*p* < 0.01). As the data were analyzed separately for each production period, these comparisons do not constitute a formal test of the treatment × period interaction.

### 3.2. Egg Quality

Egg quality parameters are shown in Table 4. At week 4, eggs from the OUHW group exhibited significantly higher eggshell compressive elastic deformation, eggshell brittle deformation, egg weight, yolk color, and eggshell thickness than those from the CG (*p* < 0.05). The greater brittle fracture deformation indicates a change in the deformation behavior of the eggshell at fracture rather than, by itself, an improvement in overall shell quality. Meanwhile, no significant differences were observed between the two groups for deformation under 9.8 N, eggshell strength, albumen height, Haugh unit, eggshell weight, yolk weight, eggshell proportion, yolk proportion, and egg shape index (*p* > 0.05). At week 8, OUHW supplementation significantly increased the eggshell compressive elastic deformation, albumen height, Haugh unit, and egg shape index (*p* < 0.05) but decreased the eggshell ratio (*p* < 0.01). However, no significant differences were detected between the CG and OUHW groups for deformation under 9.8 N, eggshell strength, eggshell brittle-fracture deformation, egg weight, yolk color, eggshell weight, yolk weight, yolk proportion, and eggshell thickness (*p* > 0.05). At week 11, eggshell deformation, albumen height, Haugh unit, and eggshell thickness were significantly increased in the OUHW group compared with the CG (*p* < 0.05). However, no significant differences were observed between groups for eggshell compressive elastic deformation, eggshell strength, eggshell brittle fracture deformation, egg weight, yolk color, eggshell weight, yolk weight, eggshell percentage, and yolk percentage (*p* > 0.05).

### 3.3. Amino Acid Composition of Egg

At week 6, the OUHW group had significantly higher concentrations of serine, glutamic acid, glycine, alanine, cysteine, histidine, and total umami amino acids in eggs compared with the CG (*p* < 0.05), whereas the concentrations of valine and isoleucine were significantly lower (*p* < 0.05; Table 5). By week 11, no significant differences in amino acid profiles were observed between the two groups (*p* > 0.05).

### 3.4. Fatty Acid Composition of Egg

Egg fatty acid composition is presented in Table 6. The contents of C17:0, C18:0, C18:1n-9, C20:4n-6, C22:6n-3, and total fatty acids were significantly higher at week 6 (*p* < 0.05), whereas the C18:3n-3 content was significantly lower in the OUHW group compared with the CG (*p* < 0.05). C20:4n-6 is a precursor of multiple lipid mediators with distinct biological functions, the biological significance of the increased C20:4n-6 level should be interpreted with caution. No significant differences between the two groups were observed in terms of the contents of C14:0, C14:1, C15:0, C16:0, C16:1, C18:2n-6c, C20:0, C18:3n-6, C20:1, C20:2, C22:0, C20:3n-6, or C22:1n-9 (*p* > 0.05). At week 11, only the C22:0 content was significantly elevated in the OUHW group (*p* < 0.05). No significant differences between the two groups were detected for C14:0, C14:1, C15:0, C16:0, C16:1, C17:0, C18:0, C18:1n-9c, C18:2n-6c, C20:0, C18:3n-6, C18:3n-3, C20:1, C20:2, C20:3n-6, C22:1n-9, C20:4n-6, C24:0, C24:1, C22:6n-3, or total fatty acids (*p* > 0.05).

### 3.5. Serum Biochemical Parameters

The effects of OUHW on serum reproductive hormones, antioxidant capacity, inflammatory cytokines, and immune-related indices are shown in Table 7. Serum FSH, LH, and E2 concentrations were significantly higher in the OUHW group than in the CG (*p* < 0.05), whereas PROG concentration did not differ between the two groups (*p* > 0.05). Regarding antioxidant capacity, OUHW supplementation significantly increased serum T-AOC, GSH concentration, and the activities of GSH-Px, SOD, and CAT, while significantly decreasing MDA concentration compared with the CG (*p* < 0.05). For inflammatory cytokines, serum IL-1β, IL-6, and TNF-α concentrations were significantly lower, whereas the IL-10 concentration was significantly higher in the OUHW group than in the CG (*p* < 0.05). Regarding immune-related indices, no significant difference in serum IgA concentration was observed between the two groups (*p* > 0.05), whereas IgG and IgM concentrations were significantly higher in the OUHW group than in the CG (*p* < 0.05).

### 3.6. Serum Metabolomic Profiles

**Figure 1:**
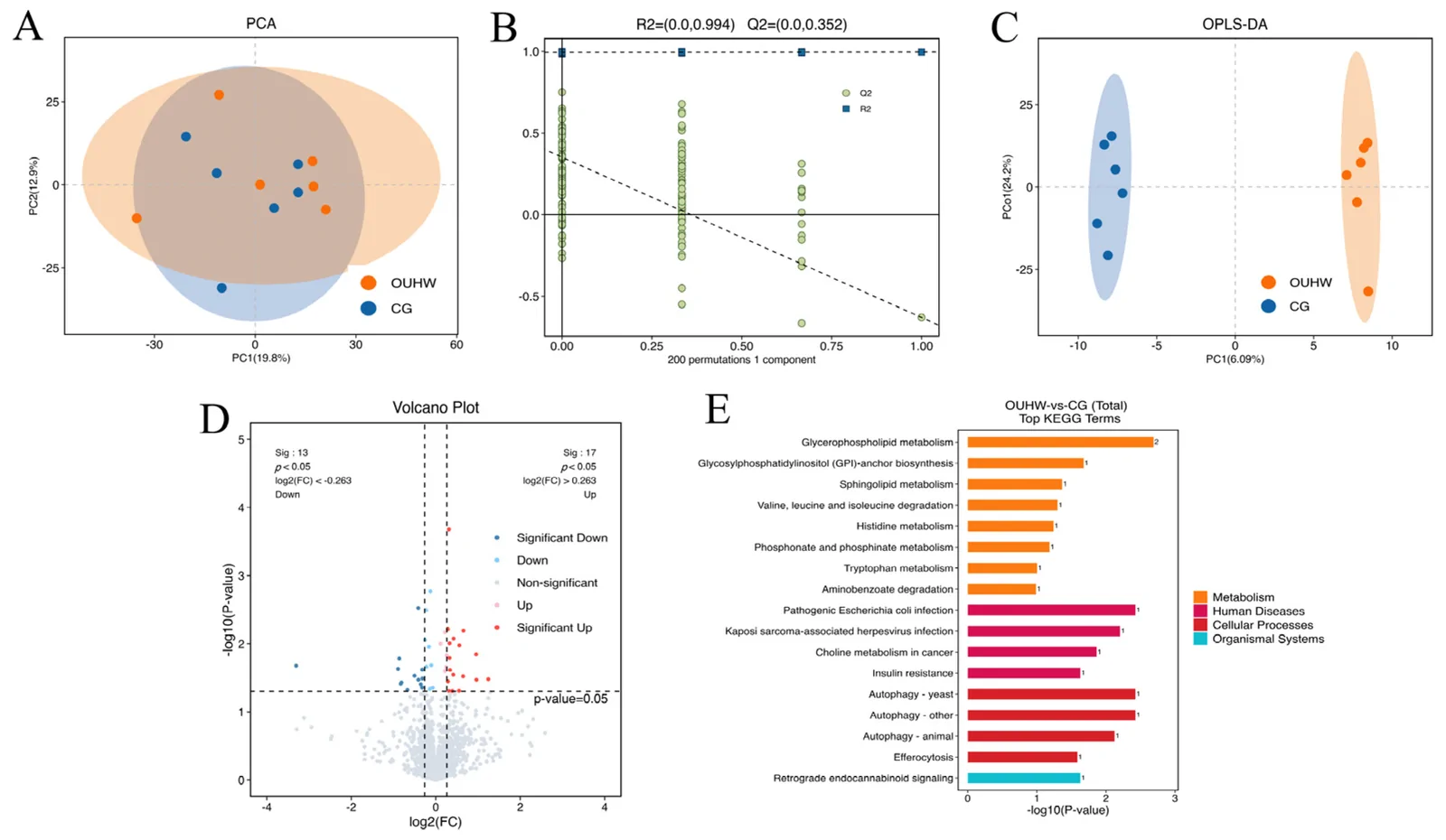
Effect of OUHW on the serum metabolome of late-laying hens (*n* = 6). (**A**): PCA score plot; (**B**): Permutation test; (**C**): OPLS-DA score plot; (**D**): Volcano plot; (**E**): KEGG pathway enrichment analysis. CG: drinking normal water; OUHW: drinking oligomeric ultrafine-nano hydrogen water.

Principal component analysis (PCA) showed substantial overlap between the CG and OUHW groups, with PC1 and PC2 explaining 19.8% and 12.9% of the total variance, respectively (Figure 1A). In contrast, the supervised OPLS-DA model showed discrimination between the two groups (Figure 1C), and the model was evaluated using a 200-permutation test (Figure 1B). Based on the criteria of VIP > 1.0 and nominal *p* < 0.05, 30 candidate differential metabolites were identified, including 17 upregulated and 13 downregulated metabolites (Figure 1D). Based on the thresholds of VIP > 1.0 and *p* < 0.05, 30 significantly differential metabolites were identified (Table S2). Specifically, 17 metabolites were upregulated in the OUHW group, including 4,7-dihydroxycoumarin, N-docosahexaenoyl lysine, phosphonoacetic acid, Pro-Leu-Gln, acetyl-L-carnitine, and hydroxybutyrylcarnitine, among others (*p* < 0.05). Conversely, 13 metabolites, such as N-acetylvaline, 3-hydroxyisovaleric acid, dipfluzine, and 12-aminododecanoic acid, were significantly downregulated in the OUHW group (*p* < 0.05). KEGG enrichment analysis revealed that these differential metabolites were primarily enriched in pathways associated with amino acid metabolism, lipid metabolism, energy metabolism, and antioxidant responses.

### 3.7. Cecal Metabolites

The cecal concentration of p-Cresol was significantly lower in the OUHW group compared with the CG (*p* < 0.05; Table 8). No significant differences were detected in the levels of phenol, indole, 3-methylindole, or ammonium nitrogen (*p* > 0.05). Among the short-chain fatty acids, the concentrations of acetic acid and isovaleric acid were significantly higher in the OUHW group than in the CG (*p* < 0.05), whereas no significant differences were observed in the concentrations of propionic acid, n-valeric acid, n-butyric acid, isobutyric acid, or hexanoic acid between the two groups (*p* > 0.05).

### 3.8. Inflammatory Cytokines and Mucosal Immune Indices

**Figure 2:**
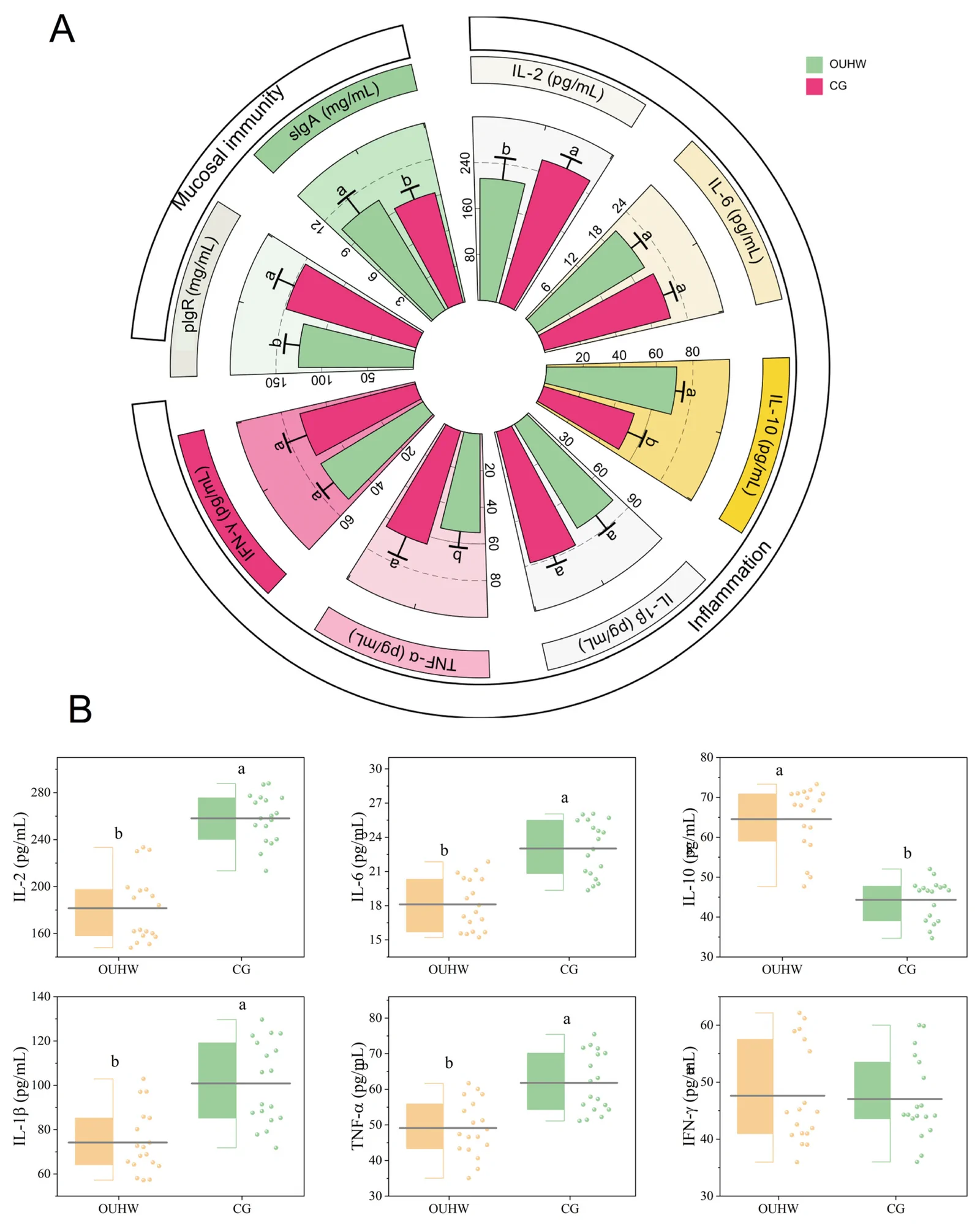
Effects of OUHW on inflammatory factors and immunity in the jejunum (**A**) and oviductal isthmus (**B**) of late-laying hens (*n* = 6). CG: drinking normal water; OUHW: drinking oligomeric ultrafine-nano hydrogen water. Different lowercase letters indicate significant differences among groups (*p* < 0.05).

The effects of OUHW on inflammatory cytokines and immune parameters in the oviductal isthmus and jejunum are presented in Figure 2. In the jejunal mucosa, the concentration of IL-10 was significantly elevated (*p* < 0.05), and IL-2 and TNF-α was decreased (*p* < 0.05) in the OUHW group. Furthermore, the level of sIgA was significantly increased (*p* < 0.05), whereas the pIgR level was decreased (*p* < 0.05, Figure 2A).

In the oviductal isthmus, as shown in Figure 2B, OUHW supplementation significantly decreased the concentrations of the pro-inflammatory cytokines IL-2 and TNF-α (*p* < 0.05), while increasing the anti-inflammatory cytokine IL-10 (*p* < 0.05).

### 3.9. Intestinal and Oviductal Microbiota

**Figure 3:**
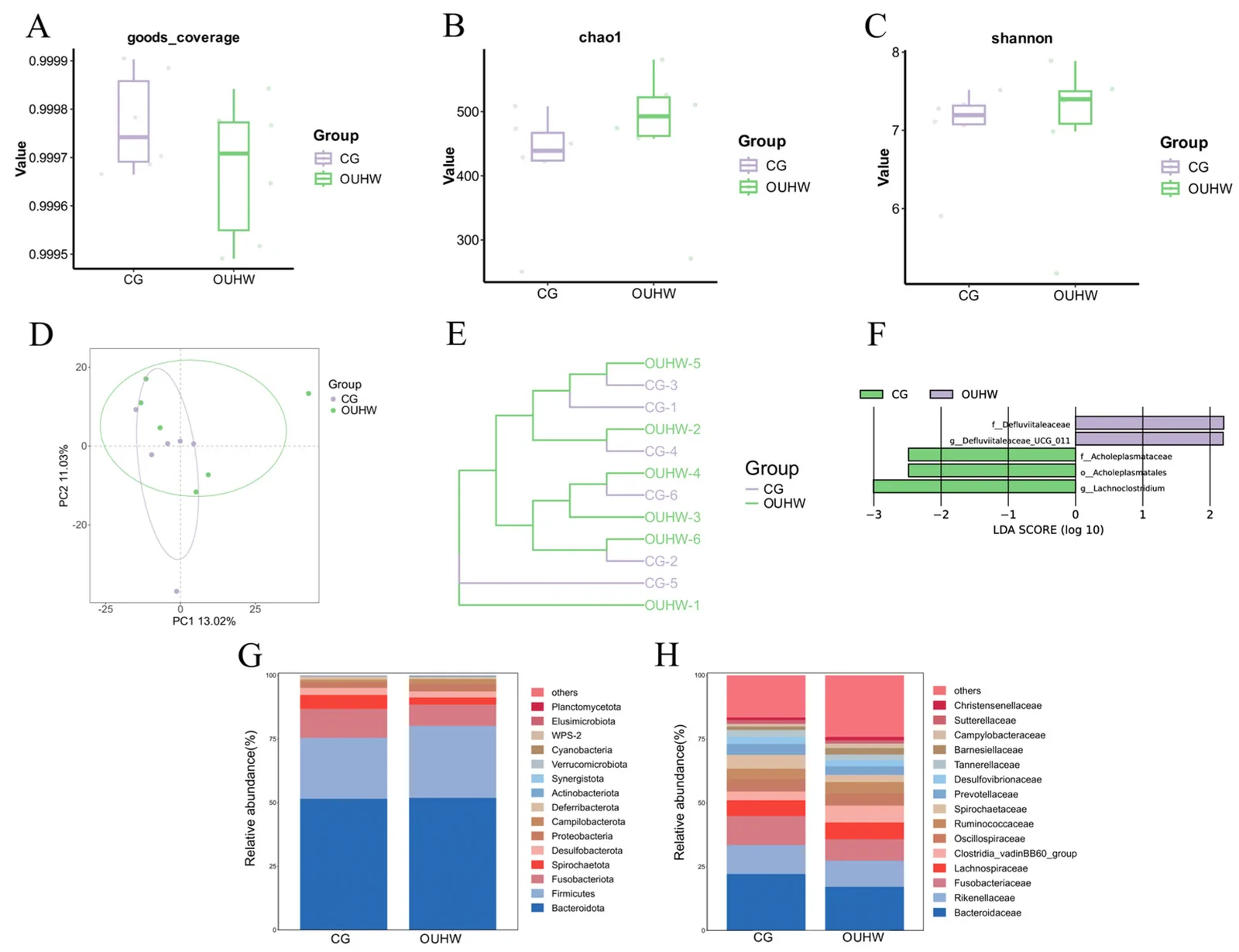
Effect of OUHW on cecal microbiota structure in late-laying hens (*n* = 6). (**A**) Good’s coverage index of samples in each treatment group; (**B**) Chao1 index; (**C**) Shannon index; (**D**) PERMANOVA analysis; (**E**) UPGMA hierarchical clustering analysis based on Bray–Curtis distance; (**F**) Analysis of differential microbiota screened by linear discriminant analysis effect size (LEfSe); Relative abundance composition of the cecal microbiota at the phylum (**G**) and genus (**H**) levels across different treatment groups.

**Figure 4:**
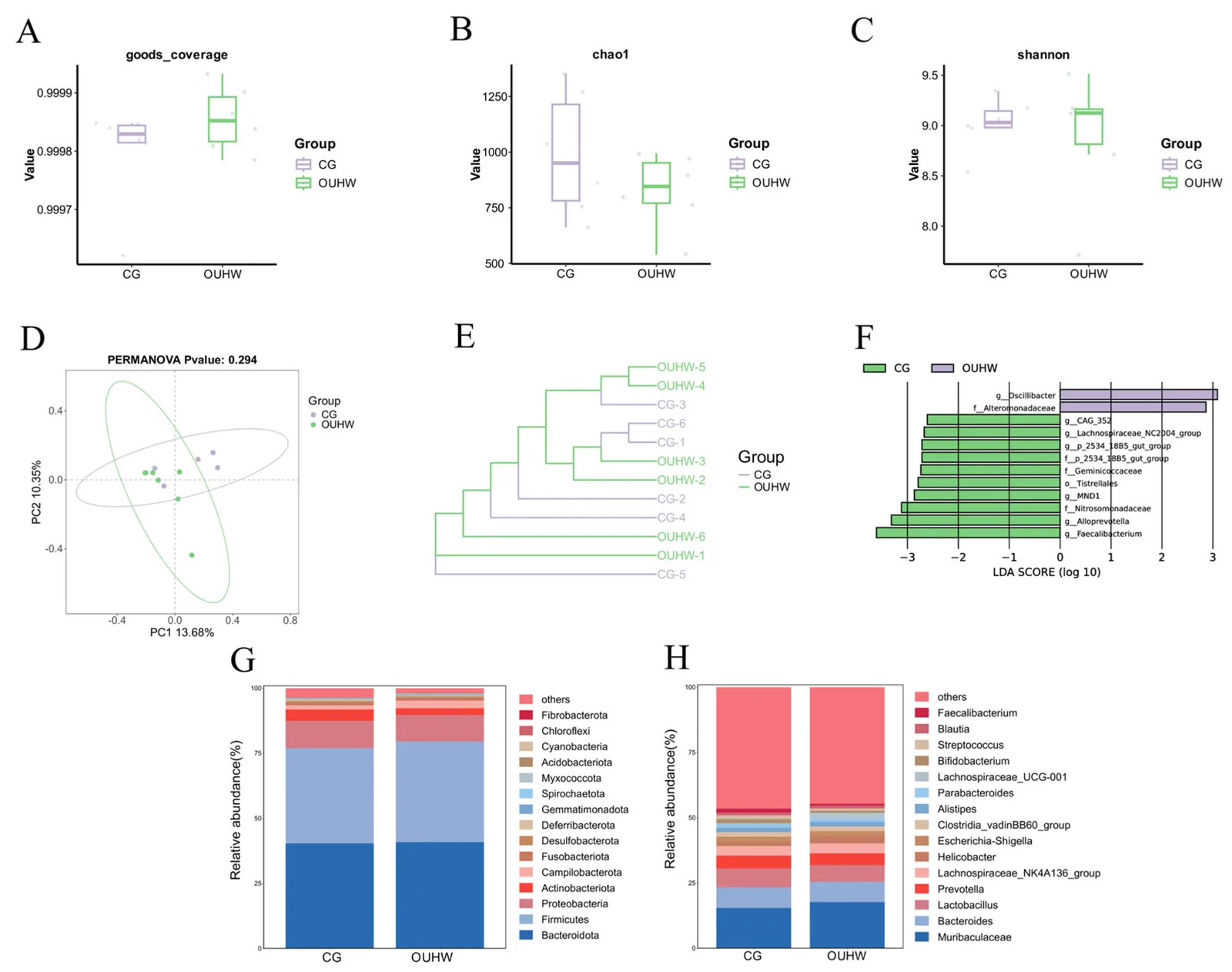
Effect of OUHW on oviductal microbiota structure in late-laying hens (*n* = 6). (**A**) Good’s coverage index of samples in each treatment group; (**B**) Chao1 index; (**C**) Shannon index; (**D**) PERMANOVA analysis; (**E**) UPGMA hierarchical clustering analysis based on Bray–Curtis distance; (**F**) Analysis of differential microbiota screened using linear discriminant analysis effect size (LEfSe); Relative abundance composition of the oviductal microbiota at the phylum (**G**) and genus (**H**) levels across different treatment groups.

High-throughput 16S rRNA gene sequencing was performed to analyze the microbiota structure of the intestinal (Figure 3) and oviduct mucosa (Figure 4). The Good’s coverage index was >0.99 for all samples, indicating sufficient sequencing depth (Figure 3A and Figure 4A).

No significant differences were observed in the α-diversity of cecal microbiota (Chao1 and Shannon indices, Figure 3B,C) between the two groups (*p* > 0.05). PCA and cluster analysis showed a certain separation trend between the OUHW group and the CG group, indicating that OUHW can reshape the composition of cecal microbiota to a certain extent (Figure 3D,E). LEfSe analysis further indicated that the OUHW group enriched microbial communities such as *Acholeplasmatales* and *Lachnoclostridium* (Figure 3F), At the phylum level, OUHW mainly maintained the overall stability of cecal microbiota (Figure 3G) and promoted the increase in fermentation metabolic genera at the genus level (Figure 3H). These results suggested that OUHW may promote the proliferation of beneficial bacteria associated with short chain fatty acids, thereby optimizing the gut microbiota environment.

In contrast, the effect of OUHW on oviduct microbiota was relatively limited. PCA and cluster analysis did not show a clear separation between the OUHW group and the CG group (see Figure 4D,E), indicating that OUHW did not markedly alter the overall community structure of oviduct microbiota. Nevertheless, LEfSe analysis identified several differential taxa in the OUHW group, including *Lachnospiraceae*-related taxa, whereas the CG group was enriched in the genera *Oscillibacter* and *Alteromonadaceae* (Figure 4F). Similarly to the cecal microbiota, OUHW mainly maintained the overall stability of oviduct microbiota at the phylum level (Figure 4G). At the genus levels, it tended to enrich certain potentially beneficial taxa and reduce the relative abundance of several miscellaneous genera (Figure 4H). Collectively, these results indicated that OUHW exerted a more pronounced modulatory effect on cecal microbiota than on oviduct microbiota, and that its influence on the oviduct microbial community was characterized more by the selective optimization of specific beneficial taxa than by broad community remodeling.

### 3.10. Intestinal and Oviduct Morphology

**Figure 5:**
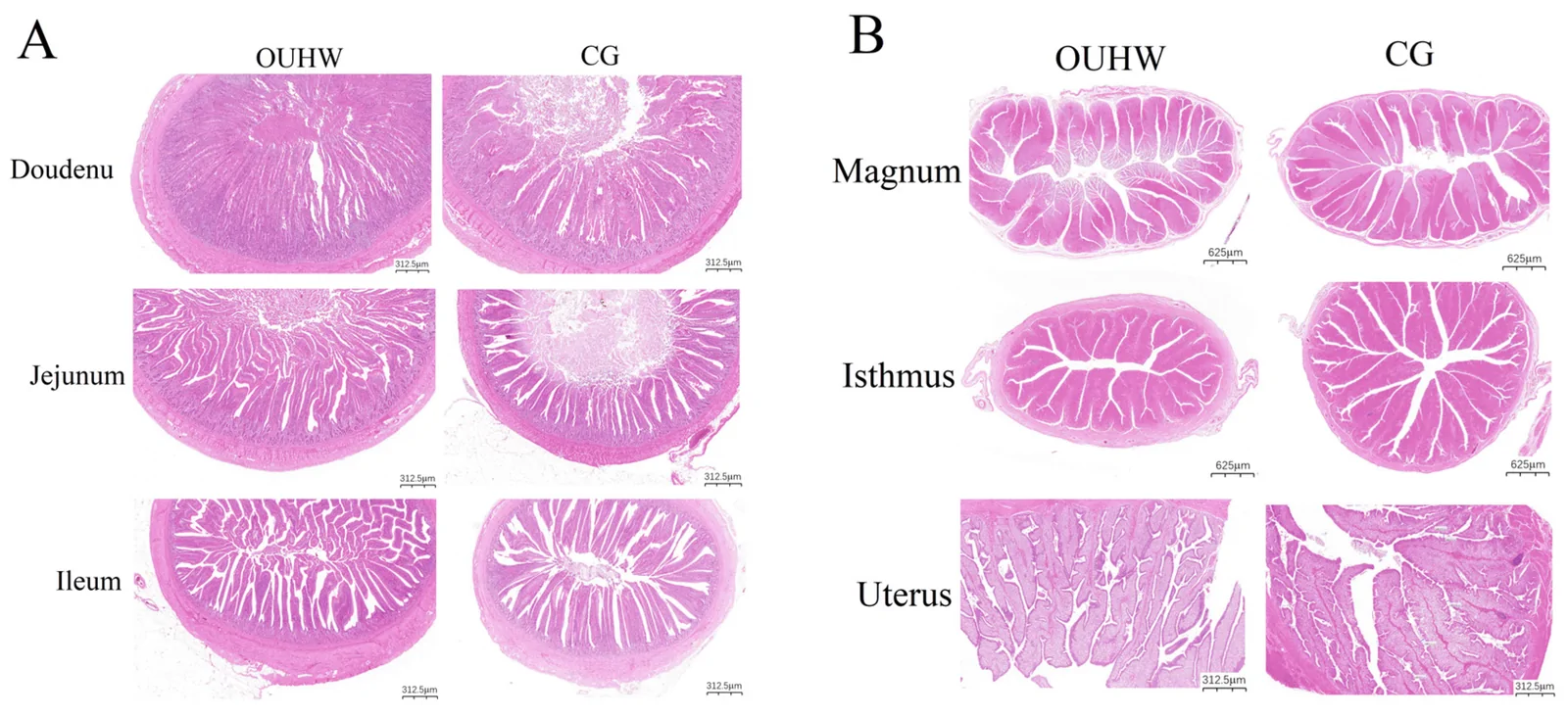
Effect of OUHW on the morphology of (**A**) small intestine (scale bar, 312.5 μm) and (**B**) oviductal morphology (scale bar, 625 μm) in late-laying hens. HE staining was performed on the duodenum, jejunum, ileum, magnum, isthmus, and uterus (*n* = 6). CG: drinking normal water; OUHW: drinking oligomeric ultrafine-nano hydrogen water.

There were no significant differences in villus height, crypt depth, or the villus height-to-crypt depth ratio in the duodenum, jejunum, and ileum between the two groups (*p* > 0.05; Table 9). Similarly, no significant differences were observed in the heights of the primary and secondary mucosal folds in the magnum, mucosal fold height in the isthmus, or interfold distance in the uterus between the two groups (*p* > 0.05; Table 10). Histological evaluation further showed that the mucosal architecture of both the intestinal segments (Figure 5A) and oviductal segments (Figure 5B) was well preserved in both groups. The intestinal villi and oviductal mucosal folds exhibited intact epithelial lining and normal morphological organization, with no evident histopathological lesions.

### 3.11. Gene Expression in Ovarian and Jejunal Tissues

**Figure 6:**
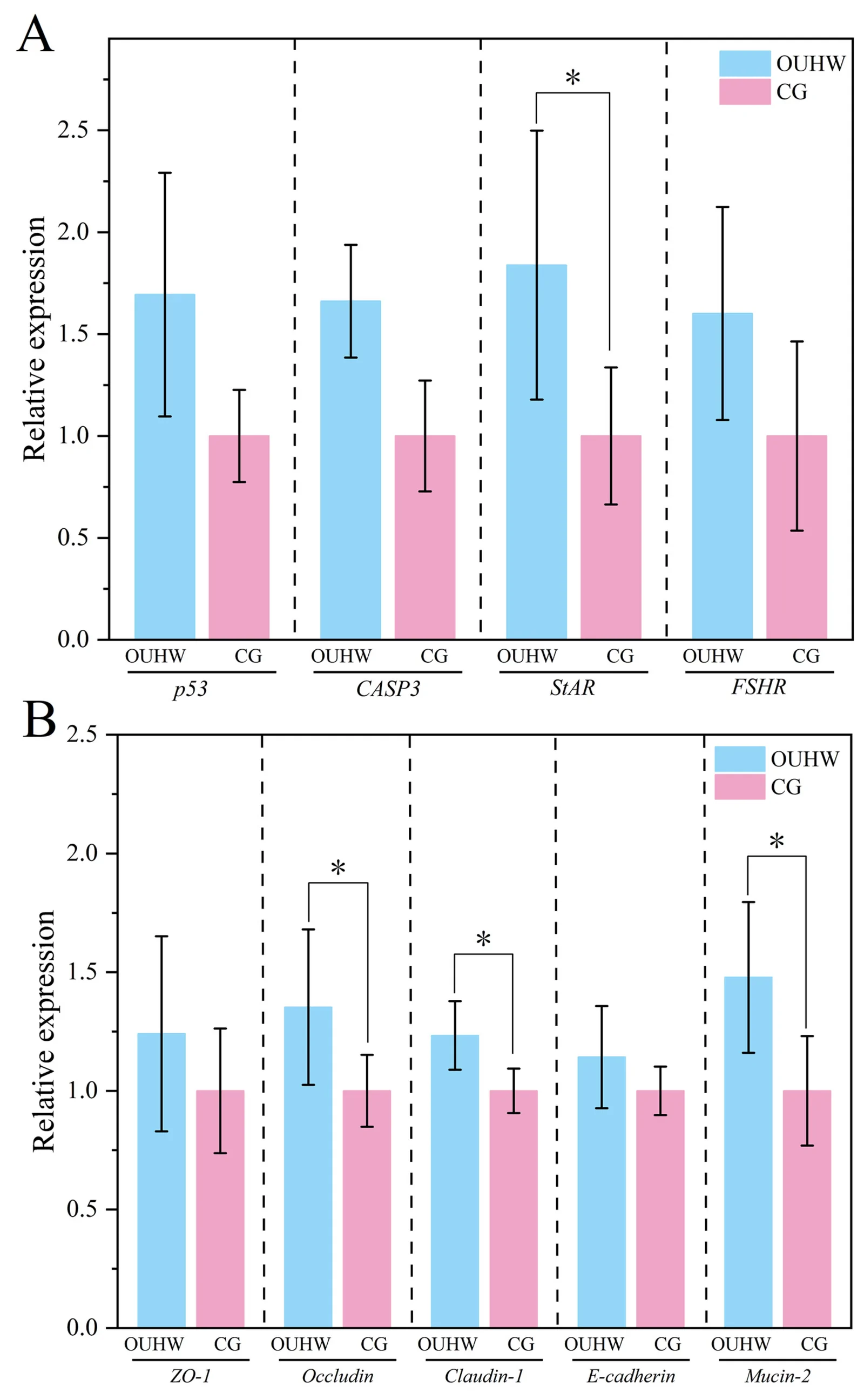
Effect of OUHW on the relative gene expression in the ovarian (**A**) and jejunal tissues (**B**) of late-laying hens. Data are presented as the mean ± SD (*n* = 6). Asterisks (“*”) indicate significant differences *p* < 0.05 compared with the CG group. CG: drinking normal water; OUHW: drinking oligomeric ultrafine-nano hydrogen water.

OUHW showed differential regulatory effects on ovarian- and intestine-related genes (Figure 6). In the ovary, no significant differences were observed in the expression levels of *P53*, *CASP3*, or *FSHR* between the OUHW and CG groups (*p* > 0.05; Figure 6A), whereas the expression levels of *StAR* were significantly upregulated in the OUHW group (*p* < 0.05, Figure 6A). In the intestine, the expression levels of *ZO-1* and *E-cadherin* did not differ significantly between the two groups (*p* > 0.05; Figure 6B), while *Occludin*, *Claudin-1*, and *Mucin-2* were significantly increased in the OUHW group compared with the CG (*p* < 0.05, Figure 6B). These results suggest that OUHW enhanced ovarian steroidogenesis-related gene expression and improved intestinal barrier function by upregulating selected tight junction- and mucus-related genes.

### 3.12. Correlation Analysis Between Serum Differential Metabolites and Differential Intestinal Flora

**Figure 7:**
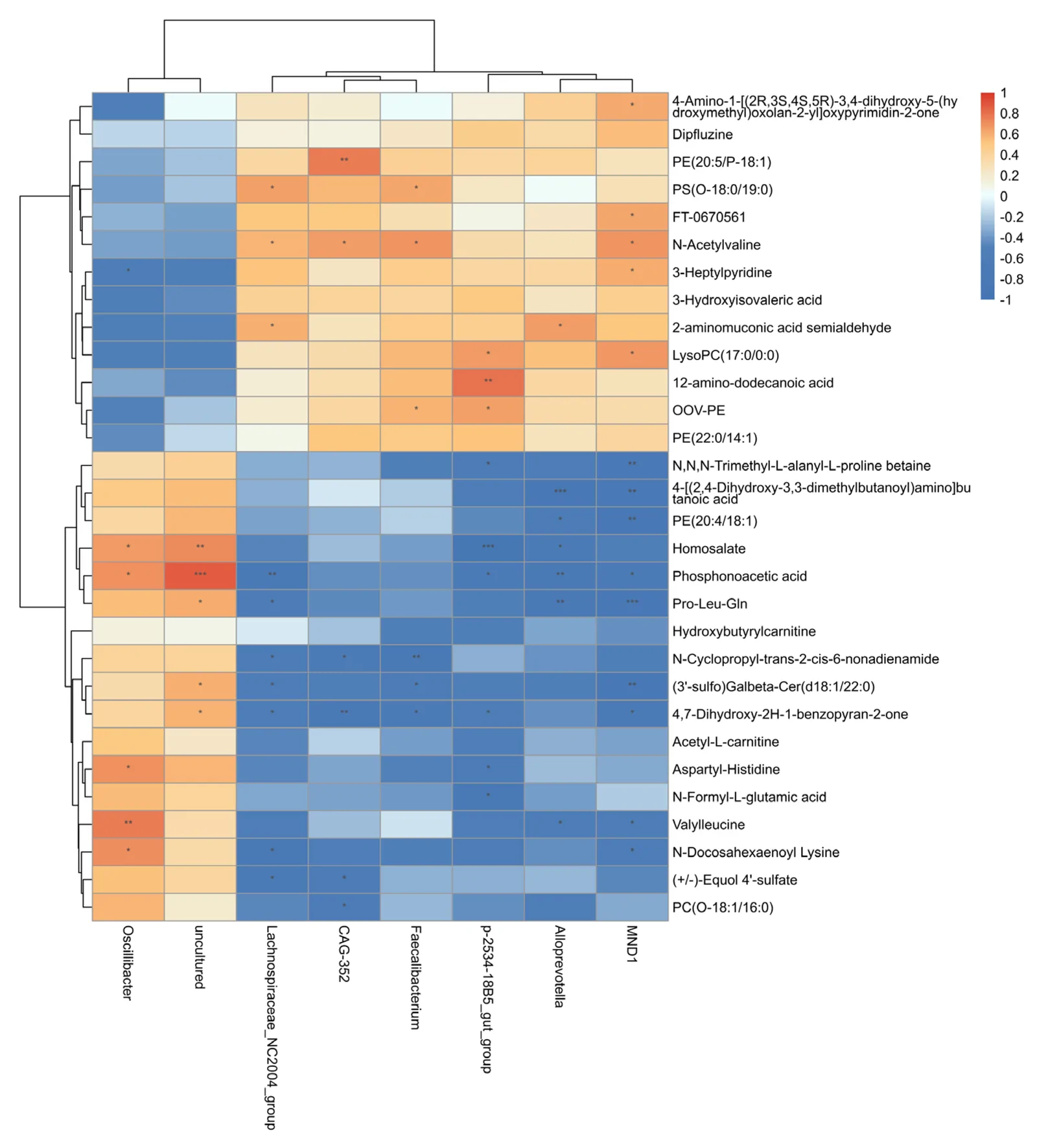
Spearman correlation analysis between characteristic intestinal genera and differential serum metabolites in the CG and OUHW groups. The color scale in the heatmap represents the Spearman correlation coefficient, where red indicates a positive correlation and blue indicates a negative correlation. Asterisks “*”, “**” and “***” denote statistical significance at *p* < 0.05, *p* < 0.01, and *p* < 0.001, respectively. The horizontal axis represents the differential microorganisms at the genus level, and the vertical axis represents the differential metabolites. CG: drinking normal water; OUHW: drinking oligomeric ultrafine-nano hydrogen water.

Spearman correlation analysis revealed distinct associations between the differential intestinal microbiota and serum metabolites (Figure 7). *Oscillibacter* and the uncultured taxon showed similar correlation patterns and were generally positively associated with metabolites in the lower cluster but negatively associated with those in the upper cluster. Specifically, *Oscillibacter* was positively correlated with phosphonoacetic acid (*R* = 0.70) and Valylleucine (*R* = 0.76) but negatively correlated with 3-Heptylpyridine (*R* = −0.59), while the uncultured taxon showed a strong positive correlation with phosphonoacetic acid (*R* = 0.87) (all *p* < 0.05). In contrast, Lachnospiraceae_NC2004_group, CAG-352, Faecalibacterium, p-2534-18B5_gut_group, Alloprevotella, and MND1 exhibited largely opposite correlation patterns. Among these associations, CAG-352 was positively correlated with PE (20:5/P-18:1) (*R* = 0.77), p-2534-18B5_gut_group was positively correlated with 12-amino-dodecanoic acid (*R* = 0.79), and MND1 was positively correlated with N-acetylvaline (*R* = 0.69) and LysoPC (17:0/0:0) (*R* = 0.68) (all *p* < 0.05). Conversely, p-2534-18B5_gut_group was negatively correlated with homosalate (*R* = −0.88), Alloprevotella was negatively correlated with 4-[(2,4-dihydroxy-3,3-dimethylbutanoyl) amino]butanoic acid (*R* = −0.84), and MND1 was negatively correlated with Pro-Leu-Gln (*R* = −0.81) and PE (20:4/18:1) (*R* = −0.80) (all *p* < 0.05). Collectively, these associations involved metabolites related to phospholipid, amino acid/peptide, and acylcarnitine metabolism, indicating close associations between OUHW-associated alterations in the intestinal microbiota and changes in the serum metabolic profile.

## 4. Discussion

HRW has been reported to exert selective antioxidant and anti-inflammatory effects in animals and humans [14]. However, conventional HRW is often limited by low hydrogen solubility, rapid hydrogen dissipation, and poor storage stability, which may restrict its biological efficacy. In the present study, OUHW was prepared using a physical approach that simultaneously reduced the size of water molecular clusters and generated ultrafine hydrogen nanobubbles. Micro-nano hydrogen bubble technology effectively addresses the issues of the low solubility and rapid escape of hydrogen in aqueous solutions, significantly enhancing the stable presence of hydrogen in water. The hydrogen molecule itself is a highly efficient selective antioxidant capable of specifically scavenging harmful hydroxyl radicals (OH). Recent studies have shown that hydrogen nanobubbles exhibit optimal stability under neutral pH conditions (pH 6–7), while extreme acidic or alkaline environments markedly accelerate their collapse [30]. These physicochemical characteristics may partly explain the biological effects observed in late-phase laying hens. The excellent stability of OUHW nanobubbles effectively prolongs the hydrogen retention time in drinking water, ensuring a sustained and continuous hydrogen supply for late-phase laying hens. Mechanistically, bioavailable hydrogen derived from stable nano-bubble hydrogen water upregulates the activities of antioxidant enzymes (SOD, GSH-Px) to alleviate systemic oxidative stress. Meanwhile, hydrogen supplementation can mitigate intestinal inflammatory responses and maintain the expression of intestinal tight junction proteins (Occludin, ZO-1), thereby preserving intestinal barrier integrity. In addition, hydrogen can modulate the composition and structure of gut microbiota to enrich beneficial bacteria. These synergistic regulatory effects collectively improve the physiological health and production performance of aging laying hens.

The ^17^O-NMR half-peak width of OUHW was 52.08 Hz, which was markedly lower than that of conventional water in the control group. This result indicates that OUHW contained smaller water molecular clusters. Smaller or less extensively hydrogen-bonded water assemblies may exhibit greater molecular mobility, and disruption of water-cluster structures has been associated with enhanced diffusion and permeation under confined conditions [31,32]. Such physicochemical properties could theoretically facilitate mass transfer in aqueous environments, although their contribution to nutrient transport in vivo remains to be established. In addition, hydrogen in OUHW was stably dispersed as ultrafine nanobubbles with a particle size of 30–60 nm. The initial dissolved hydrogen concentration reached 1384 ppb and remained at approximately 1130 ppb after 8 h, suggesting substantially improved hydrogen retention compared with conventional hydrogen-rich water.

The prolonged stability of hydrogen nanobubbles may be partly explained by interfacial electrostatic effects, as molecular dynamics simulations suggest that an electrical double layer formed by interfacial water reorientation can partially counteract the high Laplace pressure and thereby contribute to nanobubble stability [33]. Moreover, the presence of nanobubbles has been reported to reduce the viscosity of water by 6–27% [33], potentially enhancing its biological mobility.

At the microstructural level, oligomeric water structures may contribute to nanobubble stability. A two-dimensional interfacial layer consisting of three-, four-, and five-membered ring clusters of water molecules has been identified at nanobubble interfaces, suggesting that an organized hydrogen bond network may help stabilize the gas–liquid interface [34]. This structure forms a distinctive hydrogen-bonding network that plays a critical role in the ultra-high stability of the bubbles [34]. The OUHW used in this study, prepared via water molecule depolymerization technology, achieves high hydrogen concentrations and a ^17^O NMR half-height width of ≤85 Hz, demonstrating excellent stability and molecular activity [18]. The reduced surface tension of OUHW may further enhance wettability and tissue permeability. Therefore, the combination of small-cluster water and stable hydrogen nanobubbles provides a plausible physicochemical basis for the improved antioxidant status, immune regulation, nutrient deposition, and productive performance observed in this study.

Production performance is a key indicator for evaluating nutritional and drinking-water interventions in laying hens, particularly during the late laying period when reproductive efficiency and egg quality gradually decline. In the present study, OUHW reduced the FCR and the incidence of manure-stained, pimpled, and cracked eggs, while increasing the laying rate, qualified egg rate, and total egg number. These effects were more evident during weeks 5 to 11 than during the early stage of the trial, suggesting that the response to OUHW may require a period of continuous intake. This positive outcome reflects the synergistic action of oligomeric water and micro-nano hydrogen bubbles. Notably, in the biomedical field, ultra-fine bubble hydrogen water (UBHW) prepared using this technology has demonstrated remarkable biological protective effects. For instance, in mouse models, UBHW significantly increased survival rates following sub-lethal and lethal doses of radiation [35]. In this study, similar improvements in laying performance have been reported in heat-stressed hens receiving hydrogen-rich water [20]. Although those previous studies confirmed the biological functions of conventional hydrogen-rich water, the OUHW used in our work possesses superior hydrogen-retention stability compared with conventional hydrogen-rich water, which enables longer-lasting hydrogen supply. Notably, the present experiment was conducted under normal temperature conditions, indicating that OUHW may exert beneficial effects beyond stress-relief scenarios. The improved stability and sustained hydrogen availability of OUHW may contribute to these more persistent responses. To our knowledge, this study represents the first report of applying OUHW to late-laying hens, demonstrating its promising prospects for practical livestock application.

Eggshell quality declines as the laying cycle progresses, mainly due to reduced calcium deposition capacity, oxidative damage, and chronic inflammation in the reproductive tract [36,37]. In this study, OUHW improved multiple egg quality traits, including eggshell thickness, eggshell mechanical deformation indices, albumen height, and Haugh unit. These changes were accompanied by increased serum SOD activity and decreased MDA concentration, indicating improved systemic antioxidant status and reduced lipid peroxidation. Oxidative stress is known to impair epithelial cell function and mineral transport in the oviduct, thereby compromising eggshell formation [38,39]. The reduction in pro-inflammatory cytokines IL-2 and TNF-α and the increase in anti-inflammatory cytokine IL-10 in the oviductal isthmus further suggest that OUHW alleviated local inflammatory responses in the reproductive tract. Excessive uterine inflammation has been reported to impair eggshell mineralization by disrupting Ca^2+^ and HCO_3_^−^ transport and the expression of eggshell matrix proteins, thereby reducing eggshell thickness and mechanical strength [37]. Improved oviductal antioxidant and inflammatory status has also been associated with better eggshell quality in late-phase laying hens [40]. However, because eggshell calcification occurs predominantly in the uterus, whereas cytokines were measured in the isthmus in the present study, a direct causal relationship cannot be established. Thus, the improved eggshell quality may partly be associated with a more favorable oviductal redox and inflammatory environment rather than a direct effect of isthmus inflammation on eggshell mineralization.

The nutritional composition of eggs is closely related to their functional value and sensory characteristics. At week 6, OUHW increased the contents of several amino acids, including serine, glutamic acid, glycine, alanine, cysteine, and histidine, as well as total umami amino acids. Glutamic acid, glycine, and alanine are important contributors to egg flavor [26]. OUHW also increased the levels of several fatty acids, including C17:0, C18:0, C18:1n-9, C20:4n-6, C22:6n-3, and total fatty acids. In particular, docosahexaenoic acid (DHA; C22:6n-3) is an important n-3 polyunsaturated fatty acid that improves the nutritional value of eggs [41]. However, the increase in DHA was accompanied by a reduction in α-linolenic acid (C18:3n-3) and increases in the saturated fatty acids C17:0 and C18:0. Therefore, the fatty acid response to OUHW should not be interpreted solely as an improvement in the nutritional quality of the eggs. Rather, OUHW altered the deposition pattern of individual fatty acids, with increases in both potentially desirable fatty acids, such as DHA, and saturated fatty acids. The decrease in C18:3n-3 further indicates that the response of n-3 fatty acids was not uniform. Because total quantified fatty acids were also higher in the OUHW group at week 6, these differences may reflect broader changes in lipid deposition rather than selective enrichment of DHA alone. These changes were mainly observed at week 6 and became less evident at week 11, suggesting that OUHW may transiently enhance nutrient deposition during the middle phase of supplementation. Given that nutrient digestibility was not measured and intestinal morphology was unaffected by OUHW, the present study provides no direct evidence that OUHW enhanced nutrient digestion or absorption. Although hydrogen-rich water has been reported to alleviate intestinal barrier damage under challenge conditions [42], these effects do not necessarily indicate improved nutrient digestibility. Therefore, the beneficial effects of OUHW observed in the present study may be more closely associated with its antioxidant effects, whereas its potential influence on intestinal function and nutrient utilization requires further investigation.

Serum metabolomic analysis provided further insights into the metabolic responses induced by OUHW. The clear separation between the OUHW and control groups in PCA and OPLS-DA models indicated that OUHW altered the serum metabolic profile. Among the differential metabolites, acetyl-L-carnitine and hydroxybutyrylcarnitine were significantly upregulated, suggesting alterations in fatty acid oxidation and mitochondrial energy metabolism. It should be noted that elevated circulating acylcarnitines may also occur under conditions of incomplete fatty acid oxidation and metabolic stress and, therefore, could potentially be associated with hepatic lipid metabolic disturbances. However, acylcarnitine accumulation alone is not sufficient to indicate fatty liver or systemic oxidative stress, particularly in the absence of direct evidence of hepatic lipid accumulation or pathological changes. Moreover, such an adverse interpretation appears inconsistent with the improved egg quality observed in the OUHW group. Thus, the changes in these metabolites are more appropriately interpreted as reflecting a modulation in lipid and energy metabolism following OUHW treatment rather than as direct evidence of metabolic dysfunction. In addition, the upregulation of lipid-related metabolites such as PC (O-18:1/16:0) and PE (20:4/18:1), further suggests changes in phospholipid remodeling and membrane lipid metabolism. Phosphatidylcholines and phosphatidylethanolamines are important components of cell membranes and lipoproteins and are closely involved in lipid transport and metabolism [43]. Collectively, these metabolomic changes may partly explain the altered egg fatty acid profile observed in the OUHW group. However, because the metabolomic analysis was performed in serum, the physiological significance of these changes and their tissue-specific relevance to the liver, ovary, and oviduct require further verification.

Late-phase laying hens often experience chronic low-grade inflammation and immunosenescence, which can impair both intestinal and reproductive functions. Among the detected pro-inflammatory cytokines in the oviductal isthmus, OUHW produced significant reductions, specifically for IL-2 and TNF-α, with no significant alterations observed for other pro-inflammatory mediators, while increasing IL-10. This cytokine pattern indicates a shift toward an anti-inflammatory immune environment. IL-2 and TNF-α are key mediators of inflammatory injury and can negatively affect epithelial function and tissue homeostasis, whereas IL-10 is a major anti-inflammatory cytokine that limits excessive immune activation [44]. Hydrogen has been shown to selectively scavenge highly reactive oxygen species and inhibit NF-κB-mediated inflammatory signaling in rats [14] (Hu et al., 2021). The stable hydrogen release from nanobubbles in OUHW may prolong this regulatory effect in target tissues.

In the jejunum, pro-inflammatory cytokines tended to decrease but did not differ significantly between groups. However, IL-10 was significantly increased, and IL-2 and TNF-α decreased, indicating that OUHW also promoted an anti-inflammatory state in the intestinal mucosa. Moreover, OUHW increased sIgA while reducing pIgR. Secretory IgA is an important effector molecule in mucosal immunity and contributes to pathogen neutralization and intestinal barrier protection [45] (Mantis et al., 2011). pIgR mediates the transcytosis of polymeric IgA across mucosal epithelial cells, and the observed decrease in pIgR alongside increased sIgA may indicate feedback regulation or altered IgA transport, although the mechanism remains to be clarified [46]. Importantly, OUHW significantly upregulated intestinal *Occludin* mRNA expression. Occludin is a key tight junction protein involved in epithelial barrier integrity, playing a crucial role in maintaining the paracellular seal and regulating macromolecular permeability [47]. Therefore, the increase in *Occludin* expression, together with higher sIgA and IL-10 levels, suggests that OUHW primarily improved intestinal-barrier-related function through immunological and molecular regulation rather than through gross morphological remodeling.

Cecal metabolites further reflected the effects of OUHW on the intestinal microenvironment. OUHW significantly reduced cecal p-Cresol, a harmful microbial metabolite derived from protein fermentation, which can impair epithelial barrier function and exert cytotoxic and oxidative effects on intestinal epithelial cells [48]. Meanwhile, OUHW increased acetic acid and isovaleric acid concentrations. Acetate is a major microbial fermentation product that can contribute to intestinal health by serving as an energy substrate and supporting epithelial barrier integrity and immune homeostasis, while isovalerate-a branched-chain fatty acid (BCFA), generated primarily from the microbial fermentation of BCFA and elevated cecal isovalerate, is generally considered an indicator of increased proteolytic or putrefactive fermentation of undigested protein [49]. These findings suggest that OUHW altered cecal metabolite profiles and reduced potentially harmful metabolites; increased isovalerate may indicate enhanced proteolytic fermentation rather than an improved cecal environment. Further studies integrating microbiome and metabolome analyses are needed to clarify the mechanisms underlying these metabolic changes.

The 16S rRNA sequencing results showed that OUHW did not significantly affect the alpha diversity, beta diversity, or genus-level composition of the oviduct mucosal microbiota. This finding suggests that the beneficial effects of OUHW on oviductal inflammation and egg quality were not primarily mediated by large-scale remodeling of the oviduct microbiota. Instead, OUHW may act mainly through direct modulation of host redox status, inflammatory signaling, epithelial function, and metabolism. Previous human studies have also suggested that hydrogen-rich water may regulate host physiology without necessarily causing major shifts in microbial community structure [13]. The coexistence of altered cecal metabolites and unchanged oviduct microbiota structure further supports the possibility that OUHW affects microbial or host metabolic activity more than community composition. This differs from dietary additives such as prebiotics, probiotics, organic acids, and exogenous enzymes, which have been reported to reshape microbial populations more strongly in poultry [50,51], possibly because molecular hydrogen is small, diffusible, and primarily involved in redox-related signaling rather than acting as a broad antimicrobial agent.

The histological examination showed that OUHW did not significantly alter the villus height, crypt depth, or villus height-to-crypt depth ratio in the duodenum, jejunum, or ileum. Similarly, no significant changes were observed in oviductal villus-related morphological indices. These results differ from reports that hydrogen-rich water improved intestinal morphology in heat-stressed laying hens [20]. The discrepancy may be related to the physiological status of the animals. Under heat stress or pathological conditions, intestinal villi are often compromised, thereby providing greater scope for detectable morphological improvement following effective intervention [52]. In contrast, hens in the present study were maintained under normal temperature conditions, and histological sections showed intact mucosal structures with no obvious lesions. Therefore, OUHW may improve physiological function without inducing detectable structural changes. Similar observations have been reported in weanling pigs, where functional improvements in intestinal health were not necessarily accompanied by changes in villus morphology [53]. These results support the idea that OUHW acts mainly through functional regulation, including antioxidant defense, inflammatory control, metabolic modulation, and barrier-related gene expression, rather than through tissue structural remodeling.

Correlation analysis further indicated that OUHW-responsive cecal microbial taxa were closely associated with serum differential metabolites, particularly those involved in glycerophospholipid metabolism, amino acid metabolism, and acylcarnitine-related metabolism. Although OUHW did not markedly alter the overall cecal microbiota structure, these results suggest that selective modulation of specific taxa may have contributed to the systemic metabolic changes observed in serum. Such metabolic remodeling may be relevant to the significant upregulation of ovarian StAR, a key regulator of steroidogenesis, as it controls mitochondrial cholesterol transport, and lipid metabolism is therefore closely associated with steroid hormone synthesis [54]. In addition, the increased expression of jejunal Occludin, Claudin-1, and Mucin-2 may also be associated with changes in cecal microbial metabolism, as indicated by increased acetate and isovalerate and decreased p-cresol. Notably, elevated isovalerate may reflect enhanced proteolytic fermentation of undigested protein and should therefore not be interpreted as a beneficial change in the cecal environment. Together, these findings support a potential microbiota–metabolite–host interaction axis linking selective microbial modulation with improved ovarian function and intestinal-barrier-related gene expression.

## 5. Conclusions

OUHW supplementation induced specific physiological and metabolic changes in late-laying hens. OUHW significantly upregulated ovarian StAR expression and increased the jejunal expression of the intestinal-barrier-related genes *Occludin*, *Claudin-1*, and *Mucin-2*. Serum metabolomic profiling showed that OUHW altered metabolites, mainly those associated with glycerophospholipid, amino acid/peptide, and acylcarnitine metabolism, indicating a remodeling of circulating metabolic profiles. In the ceca, OUHW significantly increased acetate and isovalerate concentrations and decreased p-cresol concentration, while other measured fermentation metabolites were largely unchanged. The increase in isovalerate should be interpreted cautiously because it may reflect enhanced proteolytic fermentation of undigested protein rather than an improvement in the cecal environment. Although the overall cecal microbial community structure was not markedly altered, associations between OUHW-responsive bacterial taxa and serum differential metabolites suggest potential links between selective microbial changes and host metabolism. Collectively, these findings indicate that OUHW modulates ovarian steroidogenesis-related gene expression, intestinal-barrier-related gene expression, and specific serum and cecal metabolic characteristics in late-laying hens under the conditions of this 11-week experiment. However, the present study did not evaluate the commercial effectiveness, economic feasibility, long-term safety, or reproducibility of OUHW effects across different flocks and production conditions. Therefore, its practical applicability in commercial laying-hen production remains to be established. Further studies are required to validate these effects in larger and independent flocks over longer production periods, assess their economic and safety implications, and determine the causal relationships among intestinal microbiota, microbial metabolites, systemic metabolism, and reproductive function.

## Figures and Tables

**Table 1:** Composition and nutrient levels of basal diet (air-dry basis).

Items	Content (%)	Items ^2^	Content (%)
Corn ^3^	62.50	Metabolizable energy (MJ/kg)	11.05
Soybean meal ^3^	24.80	Crude protein (%)	16.50
Limestone	8.50	Calcium (Ca, %)	3.80
CaHPO_4_	1.80	Total phosphorus (TP, %)	0.60
Soybean oil	1.50	Lysine (Lys, %)	0.75
Premix ^1^	1.00	Methionine (Met, %)	0.35
Total	100.00	Methionine + Cysteine (%)	0.62

^1^ The premix provides the following per kg of diet: vitamin A 8000 IU; vitamin D_3_ 2500 IU; vitamin E 30 IU; vitamin K_3_ 2 mg; vitamin B_1_ 2 mg; vitamin B_2_ 6 mg; vitamin B_12_ 0.02 mg; niacin 40 mg; pantothenic acid 12 mg; folic acid 1 mg; biotin 0.1 mg; choline 500 mg; Mn 60 mg; Zn 50 mg; Fe 40 mg; Cu 5 mg; I 0.5 mg; Se 0.2 mg. ^2^ Metabolizable energy was a calculated value; all other values were measured. ^3^ Corn contained 88.0% dry-matter (DM); soybean meal (SBM) contained 43.0% crude protein (CP).

**Table 2:** Primers used for qRT-PCR in this study ^1^.

Genes	Sequence (5′–3′)	Product (bp)
*ZO-1*	F: GCCTACTGCTGCTCCTTACA	113
	R: GCGGTAAGGATGATGGCTCT	
*Occludin*	F: ATCAACGACCGCCTCAATCA	112
	R: TCCGCTTCAGGTCTTTGAGC	
*Claudin-1*	F: CTGGGTCTGGTTGGTGTGTT	167
	R: GCCACTCTGTTGCCATACCA	
*E-cadherin*	F: GAGTGTGAAGGGACAGCCAA	158
	R: TCCTTCTCCACTTTGCGTCG	
*Mucin-2*	F: TGTGTAACTGCACCAAGGCT	154
	R: TCACACTCCCAATGCCAACA	
*p53*	F: GCCGTGGCCGTCTATAAGAA	159
	R: GGTCTCGTCGTCGTGGTAAC	
*CASP3*	F: GCATATTCTACTGCTCCAGGCT	102
	R: TTTCCTGGCGTGTTCCTTCA	
*StAR*	F: CCCGAGCAGCAGGGATTTA	120
	R: GCCCTTCAGGTCGATGCTAA	
*FSHR*	F: GCAACTCAACCGCAAGGTTC	139
	R: GTGCATGGGCAAACAGATGC	
*GAPDH* ^2^	F: TGACCACTGTCCATGCCATC	135
	R: CTTTCCCCACAGCCTTAGCA	

^1^ F: forward primer; R: reverse primer. ^2^ Housekeeping gene for data normalization.

**Table 3:** Effects of OUHW on the production performance of late-phase laying hens ^1^.

Items	CG	OUHW	SEM ^2^	*p*-Value
Weeks 1–4 (67–70 weeks of age)				
Feed conversion ratio (g/g)	2.71	2.66	0.018	0.021
Manure-spotted egg rate (%)	9.83	9.23	0.252	0.198
Egg-breaking rate (%)	1.67	1.38	0.098	0.118
Pimpled egg rate (%)	2.83	2.57	0.142	0.091
Total number of eggs (count)	105.66	107.55	0.834	0.229
Average egg weight (g)	53.65	53.67	0.187	0.93
45–52 g rate (%)	26.65	29.06	0.676	0.012
52–60 g rate (%)	41.36	41.02	0.623	0.59
60–65 g rate (%)	5.22	4.68	0.25	0.254
Qualified egg rate (%)	73.22	74.76	0.82	0.313
Egg production rate (%)	73.37	74.69	0.585	0.027
Weeks 5–8 (71–74 weeks of age)				
Feed conversion ratio (g/g)	2.59	2.39	0.04	<0.001
Manure-spotted egg rate (%)	8.07	6.05	0.349	<0.001
Egg-breaking rate (%)	2.14	1.56	0.295	0.054
Pimpled egg rate (%)	2.77	2.06	0.318	0.041
Total number of eggs (count)	95.25	104.36	1.993	<0.001
Average egg weight (g)	53.62	54.12	0.417	0.233
45–52 g rate (%)	19.56	22.97	0.893	<0.001
52–60 g rate (%)	43.88	44.92	1.168	0.376
60–65 g rate (%)	7.47	7.49	0.716	0.979
Qualified egg rate (%)	70.90	75.39	1.318	0.001
Egg production rate (%)	66.15	72.47	1.359	<0.001
Weeks 9–11 (75–77 weeks of age)				
Feed conversion ratio (g/g)	2.39	2.30	0.006	<0.001
Manure-spotted egg rate (%)	6.89	5.68	0.324	0.008
Egg-breaking rate (%)	2.46	1.76	0.206	0.012
Pimpled egg rate (%)	2.59	1.86	0.293	0.063
Total number of eggs (count)	85.10	94.95	3.185	0.003
Average egg weight (g)	55.43	55.70	0.575	0.725
45–52 g rate (%)	17.48	16.90	1.393	0.677
52–60 g rate (%)	42.82	48.22	1.891	0.006
60–65 g rate (%)	11.25	10.36	0.83	0.313
Qualified egg rate (%)	71.55	75.47	2.184	0.077
Egg production rate (%)	59.09	65.94	2.216	0.003

CG: drinking normal water; OUHW: drinking oligomeric ultrafine-nano hydrogen water. ^1^ Data are presented as mean and SEM (*n* = 6 independent replicates per treatment). ^2^ SEM, standard error of the mean.

**Table 4:** Effects of OUHW on the egg quality of late-phase laying hens ^1^.

Items	CG	OUHW	SEM ^2^	*p*-Value
Week 4 (70 weeks of age)				
Eggshell compressive elastic deformation/mm	0.051	0.063	0.003	0.009
Deformation under 9.8 N/mm	160.285	160.28	8.431	1.000
Eggshell strength/N	3.19	3.11	0.254	0.834
Eggshell brittle fracture deformation/mm	0.159	0.188	0.01	0.048
Egg weight/g	50.67	53.04	1.005	0.04
Albumen height/mm	4.39	4.52	0.251	0.716
Yolk color (score)	5.33	6.00	0.269	0.04
Haugh unit	66.77	67.19	2.37	0.898
Eggshell weight/g	5.30	5.48	0.174	0.467
Yolk weight/g	14.11	14.54	0.373	0.425
Eggshell proportion	0.107	0.103	0.002	0.281
Yolk proportion	0.279	0.274	0.005	0.535
Eggshell thickness/mm	0.419	0.452	0.009	0.025
Egg shape index	1.32	1.31	0.011	0.577
Week 8 (74 weeks of age)				
Eggshell compressive elastic deformation/mm	0.06	0.08	0.004	0.016
Deformation under 9.8 N/mm	141.10	132.96	6.112	0.406
Eggshell strength/N	3.41	3.26	0.275	0.715
Eggshell brittle fracture deformation/mm	0.23	0.21	0.011	0.350
Egg weight/g	54.64	55.54	0.669	0.342
Albumen height/mm	5.48	6.70	0.206	0.001
Yolk color (score)	6.30	5.80	0.406	0.390
Haugh unit	73.22	82.76	2.214	0.011
Eggshell weight/g	5.86	5.60	0.128	0.165
Yolk weight/g	14.65	14.60	0.250	0.893
Eggshell proportion	0.11	0.10	0.002	0.003
Yolk proportion	0.26	0.26	0.005	0.458
Eggshell thickness/mm	0.46	0.45	0.007	0.355
Egg shape index	1.31	1.34	0.009	0.028
Week 11 (77 weeks of age)				
Eggshell compressive elastic deformation/mm	0.061	0.063	0.003	0.689
Deformation under 9.8 N/mm	132.39	144.88	7.025	0.049
Eggshell strength/N	3.05	3.16	0.238	0.654
Eggshell brittle fracture deformation/mm	0.198	0.197	0.009	0.94
Egg weight/g	56.01	55.74	0.727	0.712
Albumen height/mm	5.55	6.22	0.473	0.047
Yolk color (score)	6.28	6.58	0.295	0.311
Haugh unit	71.84	79.00	2.383	0.001
Eggshell weight/g	6.06	6.25	0.142	0.189
Yolk weight/g	14.92	14.63	0.392	0.478
Eggshell proportion	0.110	0.112	0.002	0.497
Yolk proportion	0.266	0.263	0.007	0.603
Eggshell thickness/mm	0.438	0.471	0.012	0.003
Egg shape index ^3^	1.30	1.32	0.016	0.038

CG: drinking normal water; OUHW: drinking oligomeric ultrafine-nano hydrogen water. ^1^ Data are presented as mean and SEM (*n* = 6 replicates per treatment). Five eggs were sampled from each replicate (30 eggs per treatment) for egg quality measurements, and the mean value of the five eggs within each replicate was used as the experimental unit for statistical analysis. ^2^ SEM, standard error of the mean. ^3^ Egg shape index = Egg long diameter/egg short diameter.

**Table 5:** Effects of OUHW on egg amino acid composition of late-phase laying hens ^1^.

Items (g/100 g)	CG	OUHW	SEM ^2^	*p*-Value
Week 6 (72 weeks of age)				
Aspartic acid (Asp)	0.99	1.00	0.007	0.697
Threonine (Thr)	0.42	0.43	0.004	0.245
Serine (Ser)	0.69	0.71	0.006	0.039
Glutamic acid (Glu)	1.27	1.35	0.01	<0.001
Glycine (Gly)	0.32	0.34	0.003	<0.001
Alanine (Ala)	0.56	0.58	0.004	0.008
Cysteine (Cys)	0.16	0.17	0.003	0.024
Valine (Val)	0.58	0.54	0.011	0.038
Methionine (Met)	0.26	0.24	0.009	0.172
Isoleucine (Ile)	0.43	0.37	0.009	0.007
Leucine (Leu)	0.78	0.77	0.008	0.545
Tyrosine (Tyr)	0.34	0.35	0.005	0.488
Phenylalanine (Phe)	0.56	0.56	0.008	0.934
Lysine (Lys)	0.64	0.65	0.005	0.253
Histidine (His)	0.22	0.23	0.003	0.038
Arginine (Arg)	0.51	0.52	0.006	0.476
Proline (Pro)	0.33	0.33	0.008	0.853
Essential amino acids ^1^	4.40	4.31	0.07	0.379
Flavor amino acids ^2^	4.16	4.31	0.05	0.060
Total ^4^	9.06	9.14	0.062	0.599
Week 11 (77 weeks of age)				
Aspartic acid (Asp)	0.80	0.84	0.036	0.484
Threonine (Thr)	0.37	0.39	0.018	0.533
Serine (Ser)	0.51	0.55	0.02	0.36
Glutamic acid (Glu)	1.00	1.05	0.041	0.497
Glycine (Gly)	0.27	0.29	0.011	0.513
Alanine (Ala)	0.50	0.52	0.023	0.643
Cysteine (Cys)	0.17	0.18	0.008	0.555
Valine (Val)	0.61	0.63	0.026	0.662
Methionine (Met)	0.27	0.28	0.015	0.7
Isoleucine (Ile)	0.46	0.47	0.021	0.787
Leucine (Leu)	0.74	0.77	0.034	0.574
Tyrosine (Tyr)	0.33	0.34	0.013	0.453
Phenylalanine (Phe)	0.57	0.58	0.026	0.77
Lysine (Lys)	0.58	0.61	0.024	0.462
Histidine (His)	0.20	0.21	0.01	0.697
Arginine (Arg)	0.46	0.49	0.021	0.464
Proline (Pro)	0.31	0.33	0.014	0.369
Essential amino acids ^3^	4.26	4.42	0.080	0.648
Flavor amino acids ^4^	3.39	3.56	0.085	0.512
Total ^5^	8.14	8.50	0.352	0.56

CG: drinking normal water; OUHW: drinking oligomeric ultrafine-nano hydrogen water. ^1^ Values are presented as mean and SEM (*n* = 6 replicates per treatment). Two eggs were sampled from each replicate (12 eggs per treatment) for egg amino acid composition analysis. ^2^ SEM, standard error of the mean. ^3^ Essential amino acids (including Thr, Val, Met, Ile, Leu, Phe, Lys, His, Arg); ^4^ Flavor amino acids (specific composition is detailed in the Materials and Methods). ^5^ The “Total” row represents the sum of all detected amino acids (g/100 g).

**Table 6:** Effects of OUHW on egg fatty acid composition of late-phase laying hens ^1^.

Item (g/100 g)	CG	OUHW	SEM ^2^	*p*-Value
At 6 weeks of the experiment (72 weeks of age)				
C14:0	0.0631	0.0666	0.000014	0.4287
C14:1	0.0117	0.0101	0.000002	0.3362
C15:0	0.0155	0.0152	0.000002	0.8624
C16:0	5.7905	6.0999	0.020996	0.0431
C16:1	0.6066	0.5455	0.002905	0.3476
C17:0	0.0333	0.0398	0.000013	0.0409
C18:0	2.3423	2.6513	0.007797	0.0021
C18:1n9c	9.2034	10.7919	0.076895	<0.0001
C18:2n6c	3.3684	3.2658	0.092173	0.7465
C20:0	0.008	0.0083	0.000001	0.5836
C18:3n6	0.0182	0.0187	0.000003	0.8012
C18:3n3	0.0773	0.0661	0.000031	0.0634
C20:1	0.059	0.063	0.000037	0.6077
C20:2	0.0477	0.0469	0.000071	0.9306
C22:0	0.0055	0.0054	0.000001	0.9025
C20:3n6	0.032	0.0308	0.000008	0.713
C22:1n9	0.0303	0.03	0.000006	0.9241
C20:4n6	0.3614	0.4435	0.00032	0.0003
C22:6n3	0.0049	0.0056	0.000001	0.2911
Total	22.2039	24.3745	0.223256	0.0014
At 11 weeks of the experiment (77 weeks of age)				
C14:0	0.0681	0.0696	0.000018	0.7902
C14:1	0.0128	0.0121	0.000003	0.74
C15:0	0.0148	0.0155	0.000006	0.7444
C16:0	6.1563	6.1647	0.128379	0.9849
C16:1	0.6742	0.6502	0.004761	0.7404
C17:0	0.0448	0.0463	0.000019	0.5827
C18:0	2.532	2.5566	0.015228	0.8611
C18:1n9c	10.5424	10.8154	0.431649	0.734
C18:2n6c	3.5849	3.7764	0.07734	0.4787
C20:0	0.0095	0.01	0.000001	0.4327
C18:3n6	0.0252	0.027	0.000022	0.5242
C18:3n3	0.0712	0.074	0.000121	0.6738
C20:1	0.0759	0.0821	0.000074	0.2547
C20:2	0.0492	0.0551	0.00004	0.1448
C22:0	0.0209	0.0242	0.000006	0.024
C20:3n6	0.0326	0.0311	0.000037	0.6407
C22:1n9	0.0216	0.0275	0.000061	0.1733
C20:4n6	0.3982	0.4086	0.000924	0.714
C24:0	0.0143	0.0145	0.000012	0.9201
C24:1	0.0849	0.0834	0.0001	0.7734
C22:6n3	0.1637	0.1811	0.000303	0.1543
Total ^3^	24.5972	25.1252	1.760929	0.722

CG: drinking normal water; OUHW: drinking oligomeric ultrafine-nano hydrogen water. ^1^ Values are presented as mean and SEM (*n* = 6 replicates per treatment). Two eggs were sampled from each replicate (12 eggs per treatment) for egg fatty acid composition analysis. ^2^ SEM, standard error of the mean. ^3^ The “Total” row represents the sum of all quantified fatty acids expressed on the same basis, rather than the proportion of total detected fatty acids. Note: C24:0 and C24:1 were determined in samples from both the 6-week and 11-week sampling points. These two fatty acids were omitted for week 6 data due to concentrations below the reliable quantification limit.

**Table 7:** Effect of OUHW on serum biochemical parameters ^1^.

Items	CG	OUHW	SEM ^2^	*p*-Value
**Antioxidant capacity**				
GSH (ng/mL)	4.48	6.73	0.49	<0.001
MDA (mmol/L)	6.19	5.58	0.27	0.049
GSH-Px (U/mL)	315.42	425.38	23.97	<0.001
CAT (U/mL)	41.84	62.20	4.44	<0.001
T-AOC (U/mL)	73.04	108.99	7.84	<0.001
SOD (U/mL)	101.27	166.09	14.13	<0.001
**Immunity**				
IgA (mg/mL)	649.59	714.58	64.86	0.340
IgM (mg/mL)	1518.91	1736.86	88.8	0.034
IgG (g/L)	13.99	15.91	0.82	0.041
**Inflammatory cytokines**				
IL-6 (pg/mL)	31.64	21.97	2.11	<0.001
IL-10 (pg/mL)	36.31	60.62	5.3	<0.001
TNF-α (pg/mL)	75.11	55.91	4.19	<0.001
IL-1β (pg/mL)	123.22	94.00	6.37	<0.001
**Reproductive hormones**				
FSH (mIU/mL)	6.94	9.97	0.66	<0.001
LH (ng/mL)	65.81	123.90	12.66	<0.001
E2 (pg/mL)	318.44	370.60	23.16	0.048
PROG (pg/mL)	8.40	8.98	0.57	0.335

CG: drinking normal water; OUHW: drinking oligomeric nano hydrogen water. ^1^ Data are presented as mean and SEM (*n* = 6 birds, with one bird sampled from each of six independent replicates). ^2^ SEM, standard error of the mean.

**Table 8:** Effect of OUHW on metabolic characteristics of cecal contents in late-laying hens ^1^.

Items		CG	OUHW	SEM ^2^	*p*-Value
NH_4_^+^-N, %		0.21	0.20	0.004	0.436
Hazardous substances (mg/kg of cecal contents)	Phenol	3.14	4.08	0.414	0.238
	p-Cresol	32.10	25.26	1.615	0.019
	Indole	6.18	6.55	0.724	0.771
	3-Methylindole	2.00	1.80	0.208	0.566
Short-chain fatty acids (mg/kg of cecal contents)	Acetic acid	1268.74	1475.82	32.87	0.002
	Propionic acid	660.94	587.31	22.6	0.083
	n-Valeric acid	43.79	38.85	4.91	0.53
	Isovaleric acid	181.87	224.90	11.76	0.024
	n-Butyric acid	562.13	535.80	16.09	0.418
	Isobutyric acid	37.42	38.70	3.93	0.843
	Hexanoic acid	10.82	11.03	0.45	0.792

CG: drinking normal water; OUHW: drinking oligomeric ultrafine-nano hydrogen water. ^1^ Data are presented as mean and SEM (*n* = 6). ^2^ SEM, standard error of the mean.

**Table 9:** Effect of OUHW on intestinal morphology in late-laying hens ^1^.

Intestinal Segment	Tissue Morphology (μm)	CG	OUHW	SEM ^2^	*p*-Value
Duodenum	Villus height	1105.65	1301.44	105.9	0.243
	Crypt depth	385.01	383.63	32.4	0.98
	Villus height-to-crypt depth ratio	3.04	3.58	0.379	0.421
Jejunum	Villus height	1094.18	1033.06	109.68	0.779
	Crypt depth	255.12	223.70	19.62	0.416
	Villus height-to-crypt depth ratio	4.63	5.34	0.44	0.456
Ileum	Villus height	848.66	815.84	30.37	0.605
	Crypt depth	208.24	177.03	15.12	0.39
	Villus height-to-crypt depth ratio	4.81	5.08	0.41	0.722

CG: drinking normal water; OUHW: drinking oligomeric nano hydrogen water. ^1^ Data are presented as mean and SEM (*n* = 6). ^2^ SEM, standard error of the mean.

**Table 10:** Effect of OUHW on oviductal morphology in late-laying hens ^1^.

Segment	Tissue Morphology (μm)	CG	OUHW	SEM ^2^	*p*-Value
Magnum	Primary villus length	3381.51	3526.58	192.48	0.585
	Secondary villus length	602.27	635.41	59.45	0.684
Isthmus	Villus length	2299.39	2220.81	206.77	0.803
Uterus	Inter-villus space	75.17	59.90	6.93	0.157

CG: drinking normal water; OUHW: drinking oligomeric nano hydrogen water. ^1^ Data are presented as mean and SEM (*n* = 6). ^2^ SEM, standard error of the mean.

## Data Availability

The data presented in this study are available from the corresponding author upon reasonable request.

## Supplementary Materials

The following supporting information can be downloaded at: https://www.mdpi.com/article/10.3390/ani16172658/s1. Table S1: Detailed information of commercial ELISA kits used in this study. Table S2: Regulation of serum differential metabolites in late-laying hens by OUHW.
