# Supplementary material for: Effects of Oligomeric Ultrafine-Nano Hydrogen Water on Laying Performance, Egg Quality, Nutrient Composition, and Intestinal and Reproductive Responses in Late-Laying Hens
Source: Animals (Basel). 2026 Aug 24;16(17):2658. doi: 10.3390/ani16172658 (PMC13564287; doi:10.3390/ani16172658)
Supplement: Supplementary file 1 [file animals-16-02658-s001.zip › animals-4490727-supplementary.pdf]

Oligomeric ultrafine-nano hydrogen water improves production performance and egg quality in late-laying hens through multiple functional modulation

Baowei Wang[1], Guangpeng Chu[1], Mengxiao Yang[2], Zhigang Fan[3], Yuanzhao Wu[3], Binghan Wang[4], Wei Lu[3], Shijie Fan[5], Ruilei Liu[5], Guiqin Wu[5], Mingai Zhang[2], Wenlei Fan[2], Tiejun Chen[1]*, Jing Wang[6]

Affiliations:

1. College of Food Science and Engineering, Qingdao Agricultural University, Qingdao 266109, China;
2. College of Animal Science and Technology, Qingdao Agricultural University, Qingdao 266109, China;
3. Qingdao Londs Technology Equipment Co., Ltd., Qingdao 266109, China;
4. Qingdao Huihe Biotechnology Co., Ltd., Qingdao 266109, China;
5. Beijing Huadu Yukou Poultry Industry Co., Ltd., Beijing 101206, China
6. Feed Research Institute of Chinese Academy of Agricultural Sciences, Beijing 132011, China

***Correspondence:**

chentiejun2009@126.com; Tel/Fax: 86-532-58957973

Table S1 Detailed information of commercial ELISA kits used in this study

Full name of kit	Cat. No.	Specification	Detection range	Sensitivity	Intra-assay CV	Inter-assay CV
Chicken follicle-stimulating hormone (FSH) ELISA Kit	TW13249	96T	1–100 mIU/mL	<0.1 mIU/mL	<8%	<10%
Chicken luteinizing hormone (LH) ELISA Kit	TW15154	96T	0.5–50 mIU/mL	<0.05 mIU/mL	<8%	<10%
Chicken superoxide dismutase (SOD, activity) ELISA Kit	TW14571	96T	10–1000 U/mL	<1 U/mL	<8%	<10%
Chicken malondialdehyde (MDA) ELISA Kit	TW14109H	96T	0.1–10 nmol/mL	<0.01 nmol/mL	<8%	<10%
Chicken total antioxidant capacity (TAOC) ELISA Kit	TW14008H	96T	0.05–5 U/mL	<0.005 U/mL	<8%	<10%
Chicken immunoglobulin G (IgG) ELISA Kit	TW15214	96T	0.1–10 mg/mL	<0.01 mg/mL	<8%	<10%
Chicken immunoglobulin A (IgA) ELISA Kit	TWE9407	96T	5–500 μg/mL	<0.5 μg/mL	<8%	<10%
Chicken immunoglobulin M (IgM) ELISA Kit	TWE8393	96T	10–1000 μg/mL	<1 μg/mL	<8%	<10%
Chicken estradiol (E2) ELISA Kit	TW13414	96T	5–500 pg/mL	<0.5 pg/mL	<8%	<10%

Full name of kit	Cat. No.	Specification	Detection range	Sensitivity	Intra-assay CV	Inter-assay CV
Chicken progesterone (P4) ELISA Kit	TW13622	96T	0.1–10 ng/mL	<0.01 ng/mL	<8%	<10%
Chicken catalase (CAT) activity ELISA Kit	TW14815	96T	1–100 U/mL	<0.1 U/mL	<8%	<10%
Chicken glutathione peroxidase (GSHPx) ELISA Kit	TW14688	96T	2–200 U/mL	<0.2 U/mL	<8%	<10%
Chicken glutathione (GSH) ELISA Kit	TW14349	96T	0.5–50 μmol/L	<0.05 μmol/L	<8%	<10%
Chicken interleukin-1β (IL-1β) ELISA Kit	TW13313	96T	10–1000 pg/mL	<1 pg/mL	<8%	<10%
Chicken interleukin-2 (IL-2) ELISA Kit	TW14456	96T	5–500 pg/mL	<0.5 pg/mL	<8%	<10%
Chicken interleukin-6 (IL-6) ELISA Kit	TW13417	96T	2–200 pg/mL	<0.2 pg/mL	<8%	<10%
Chicken interleukin-10 (IL-10) ELISA Kit	TW15248	96T	1–100 pg/mL	<0.1 pg/mL	<8%	<10%
Chicken tumor necrosis factor α (TNF-α) ELISA Kit	TW13636	96T	5–500 pg/mL	<0.5 pg/mL	<8%	<10%

Full name of kit	Cat. No.	Specification	Detection range	Sensitivity	Intra-assay CV	Inter-assay CV
Chicken interferon γ (IFN-γ) ELISA Kit	TW14462	96T	10–1000 pg/mL	<1 pg/mL	<8%	<10%
Chicken polymeric immunoglobulin receptor (pIgR) ELISA Kit	TW14198H	96T	0.1–10 ng/mL	<0.01 ng/mL	<8%	<10%
Chicken secretory immunoglobulin A (sIgA) ELISA Kit	TW15331	96T	1–100 μg/mL	<0.1 μg/mL	<8%	<10%

Table S2 Regulation of serum differential metabolites in late-laying hens by OUHW[1]

Metabolites	VIP	Average(OUHW)	Average(CG)	log2FoldChange	FoldChange	Regulation	p-value
4,7-Dihydroxy-2H-1-benzopyran-2-one	1.8122611	16.681188	16.368788	0.3123998	1.2417716	Up	0.0002094
N-Acetylvaline	1.986822	21.000041	21.413391	-0.41335	0.7508777	Down	0.0029815
N-Docosahexaenoyl Lysine	1.5909764	19.000996	18.71439	0.2866058	1.2197672	Up	0.006128
Phosphonoacetic acid	2.3770479	21.762251	21.107182	0.6550684	1.5746906	Up	0.0064426
Pro-Leu-Gln	1.8353279	16.669235	16.246535	0.4227004	1.3404342	Up	0.0083908
(3'-sulfo)Galbeta-Cer(d18:1/22:0)	1.6221855	18.208278	17.881036	0.3272416	1.2546123	Up	0.0098919
N,N,N-Trimethyl-L-alanyl-L-proline betaine	2.1125342	27.042607	26.483365	0.5592413	1.4734941	Up	0.010564
PC(O-18:1/16:0)	2.7672731	21.38133	20.424281	0.9570491	1.941335	Up	0.014245
Homosalate	1.6108034	16.480921	16.155679	0.3252417	1.2528743	Up	0.0161943
PE(22:0/14:1)	2.4904732	22.195321	23.059128	-0.863807	0.5495006	Down	0.0165451
FT-0670561	5.1254136	10.199006	13.504768	-3.305763	0.1011268	Down	0.0210367
3-Hydroxyisovaleric acid	2.70494	21.369619	22.262965	-0.893346	0.5383639	Down	0.0234185
Dipfluzine	1.5797391	19.144336	19.464694	-0.320357	0.8008714	Down	0.0240437
N-Cyclopropyl-trans-2-cis-6-nonadienamide	1.6198963	16.819816	16.482522	0.3372939	1.2633846	Up	0.0243306
Valylleucine	1.7848624	19.145427	18.729916	0.4155107	1.3337707	Up	0.0284623
12-amino-dodecanoic acid	1.9401662	21.173687	21.676946	-0.503259	0.7055112	Down	0.029517
4-[(2,4-Dihydroxy-3,3-dimethylbutanoyl)amino]butanoic acid	2.1775577	16.300163	15.65262	0.6475431	1.5664982	Up	0.0300488
3-Heptylpyridine	1.5482311	16.399798	16.7147	-0.314902	0.8039056	Down	0.0323238
(+/-)-Equol 4'-sulfate	3.1007205	19.750372	18.506095	1.2442768	2.3689977	Up	0.0330997
OOV-PE	1.6899745	16.524939	16.935643	-0.410704	0.7522563	Down	0.0337329
PE(20:4/18:1)	2.5725208	23.166017	22.202769	0.9632484	1.9496949	Up	0.0337843

Aspartyl-Histidine	1.4825871	20.218714	19.930642	0.2880718	1.2210073	Up	0.0356023
4-Amino-1-[(2R,3S,4S,5R)-3,4-dihydroxy-5-(hydroxymethyl)oxolan-2-yl]oxypyrimidin-2-one	2.3578675	20.960915	21.772385	-0.811469	0.5698012	Down	0.0369801
PS(O-18:0/19:0)	2.4931025	21.382243	22.211586	-0.829343	0.5627856	Down	0.0386587
PE(20:5/P-18:1)	1.547201	19.145952	19.500317	-0.354365	0.7822136	Down	0.0393139
LysoPC(17:0/0:0)	1.467992	18.582523	18.906082	-0.323558	0.7990964	Down	0.0440568
2-aminomuconic acid semialdehyde	2.1194191	20.13092	20.801921	-0.671001	0.6280707	Down	0.0474836
N-Formyl-L-glutamic acid	1.9629369	21.965111	21.414306	0.550805	1.4649029	Up	0.049051
Hydroxybutyrylcarnitine	1.5813009	22.343616	21.950308	0.3933081	1.3134016	Up	0.0494013
Acetyl-L-carnitine	1.4470965	28.658569	28.342767	0.3158022	1.2447036	Up	0.0496457
